# A two-stage algebraic framework for designing cryptographically strong S-boxes for secure image encryption

**DOI:** 10.1371/journal.pone.0357107

**Published:** 2026-09-15

**Authors:** Asima Razzaque, Hafiza Zara Mustafa, Dilshad Alghazzawi, Ghaliah Alhamzi

**Affiliations:** 1 Department of Mathematics and Statistics, College of Science, King Faisal University, Al-Ahsa, Saudi Arabia; 2 Department of Mathematics, Division of Science and Technology, University of Education, Lahore, Pakistan; 3 Department of Mathematics, College of Science & Arts, King Abdul Aziz University, Rabigh, Saudi Arabia; 4 Department of Mathematics and Statistics, College of Science, Imam Mohammad Ibn Saud Islamic University (IMSIU), Riyadh, Saudi Arabia; University of Anbar, IRAQ

## Abstract

The security of modern block ciphers heavily relies on the cryptographic strength of Substitution boxes (S-boxes), which introduce non-linearity and confusion. While numerous methods for S-box generation exist, there is a continuous pursuit of designs that offer a superior and balanced profile against various cryptanalytic attacks. This paper presents a new, two-step approach to the construction of highly nonlinear S-boxes, with group-theoretic underpinnings. During the first step, we use a parameterized permutation representation of the triangle group Δ(2,3,43) on the projective line over the finite field $F_{\text{2}\text{5}\text{7}}$, denoted by $P^{\text{1}}\left(F_{\text{2}\text{5}\text{7}}\right)$, to produce an initial S-box with a nonlinearity of 106. The second stage is the main innovation, in which we use a refinement process via the action of the permutation group $C_{\text{7406532}}\times C_{\text{3}}$ on this original S-box. The refinement increases the average nonlinearity to 112 and yields differential uniformity 4, linear approximation probability 0.0625, strong bit-independence behavior, and strict avalanche characteristics close to the ideal value. The proposed S-box is then incorporated into a multi-round grayscale image-encryption scheme based on fixed S-box substitution, key- and nonce-dependent position permutation, SHA-256-based keystream whitening, and a final bidirectional nonlinear diffusion layer. Experiments on Moon Surface, Airplane, and Clock images produce ciphertext entropies of 7.9968–7.9975, near-zero pixel correlations, NPCR values of 99.7040%–99.7559%, and UACI values of 33.4902%–33.6390%. Cropping and noise experiments further show exact recovery without attack and useful attack-dependent reconstruction under ciphertext degradation, particularly under mild cropping and low-density salt-and-pepper noise. These results demonstrate strong statistical security, differential sensitivity, and practical applicability for grayscale image protection.

## 1. Introduction

### 1.1. Background

Rapid developments in information and communication technologies have led to a pressing need to safeguard sensitive and personal information. Cryptography is a key factor in the privacy, integrity and secure transmission of data over digital platforms and providing access only to authorised users [1]. Cryptographic techniques have historically been employed by governments and organizations to keep secret confidential information in the eyes of their opponents. Today, billions of secure and encrypted communications are carried out on a daily basis, and serve as the foundation of security in financial transactions, healthcare systems, personal communication, multimedia data, and cloud-based infrastructures [2]. Hamad et al. proposed a hybrid security method based on RNA-sequence generation, true random number generation, XOR encryption, and codebook-guided image embedding. Their study illustrates the growing use of biologically inspired encoding and randomized key generation in secure communication [3]. Rashid et al. investigated an AI-assisted security framework in which artificial intelligence supports key generation before text encryption and steganographic embedding. This work reflects an emerging direction in adaptive cryptographic design [4]. Cryptographic systems can be categorised into symmetric-key and asymmetric-key systems. Modern symmetric encryption systems provide a single key that is used for both encryption and decryption and are more computationally efficient and practical than asymmetric algorithms. The two major classes of symmetric encryption techniques are block ciphers and stream ciphers [5].

In order to improve the security of block ciphers, many block cipher designs have been proposed based on Shannon's principles of confusion and diffusion. The difference between plaintext and ciphertext is blurred in confusion, but the statistical structure of the plaintext is diffused throughout the ciphertext to make the ciphertext unpredictable. S-boxes are the most significant nonlinear component in block ciphers that is required in generating confusion [6]. Cryptanalysis is usually based on S-box in cryptographic systems such as DES or AES. The security of the whole cryptosystem can be compromised by the flaws in the design of the S-boxes [7]. Thus, it is highly significant that cryptographically strong S-boxes can be constructed to create secure and efficient encryption algorithms.

### 1.2. Literature review

Existing S-box construction methods can be grouped broadly into four categories. Chaos-based methods generate permutations from nonlinear dynamical systems or chaotic maps. Algebraic methods use structures such as finite fields, Möbius transformations, elliptic curves, coset graphs, and group actions. Heuristic and evolutionary methods search the space of permutations using techniques such as ant-colony optimisation, evolutionary algorithms, cuckoo search, artificial bee colony optimisation, and related metaheuristics. Hybrid methods combine an algebraic or chaotic initial construction with a secondary optimisation, permutation, or post-processing stage. Every category has its own set of helpful attributes and drawbacks. Chaos-based methods can produce large families of candidates, but their behaviour in practice may be dependent on finite-precision arithmetic, choice of parameters and implementation details, and chaotic behaviour does not imply a balanced cryptographic profile. Although algebraic constructions offer a clear and repeatable mathematical structure, an unrefined algebraic S-box can have only moderate nonlinearity or differential uniformity. Although heuristic methods can be used to effectively optimise selected cryptographic scores, the stochastic nature of these methods can make exact reproduction more difficult, and the resulting S-box can be dependent on stopping conditions, objective-function weights, and random initialisation. Hybrid designs can mix the best of both approaches, but may add complexity and may be hard to identify which stage is responsible for the final improvement. The present study addresses these issues by combining a deterministic group-theoretic initial construction with an exhaustive search over a finite, explicitly defined permutation group.

Existing S-box construction methods can broadly be classified as chaos-based [8–15], algebraic [16–21], heuristic or evolutionary [22–26], and hybrid methods [27,28]. Table 1 summarises their main principles, advantages, and limitations.

**Table 1:** Categorization of existing S-box construction methods according to their underlying principles, advantages, limitations, and representative studies.

Category	Typical construction principle	Main advantage	Common limitation	Representative references in the manuscript
Chaos-based	Chaotic maps and nonlinear dynamical systems	Large candidate space and simple numerical generation	Finite-precision sensitivity and incomplete algebraic transparency	[8–15]
Algebraic	Finite fields, Möbius maps, elliptic curves, triangle groups, coset graphs	Reproducible structure and mathematical interpretation	Raw construction may not optimise all cryptographic criteria	[16–21]
Heuristic or evolutionary	Population-based or local-search optimisation	Direct optimisation of selected security metrics	Stochastic output, computational cost, and sensitivity to objective functions	[22–26]
Hybrid	Algebraic or chaotic seed followed by optimisation or post-processing	Can balance structure and numerical performance	Increased complexity and weaker attribution of improvements	[27,28] and the proposed framework

The S-box is an important part of block ciphers, and is responsible for adding nonlinearity and confusion to the cipher by obfuscating the relation between the plaintext and ciphertext. The 8×8 S-box of the AES has motivated extensive research for the development of cryptographically secure alternatives for boosting the security of modern encryption systems. Ahmad et al. [22] presented an algorithm for generating strong S-boxes with improved cryptographic properties based on ant colony optimization. Anees and Ahmed [8] proposed a design framework inspired by a Van der Pol oscillator to build better S-boxes from a statistical point of view. Picek et al. [23] optimized evolutionary algorithms by using an improved cost function to create robust S-boxes with higher efficiency. Ullah et al. [9] combined the chaotic systems with linear transformations to design the S-boxes with good statistical and algebraic properties. Razaq et al. [16] applied the parametrization method of Mushtaq to generate coset graphs corresponding to the triangle group Δ(2,3,4) and then applied symmetric-group-based permutations to obtain strong S-boxes. A chaos-based approach was presented by Ozkaynak [10], which produced S-boxes with excellent cryptographic characteristics especially in terms of XOR balance and nonlinearity. Shafique [11] introduced a cubic-logistic map based S-box for encryption of grayscale images. Artuger and Ozkaynak [27] proposed a practical post-processing method to improve the cryptographic performance of random S-boxes. Zahid et al. [24] proposed a method of evolutionary heuristics for the design of adaptive S-boxes of high nonlinearity. In [17], Mushtaq's parametrization method was used for the triangle group Δ(2,3,8) over $F_{\text{2}\text{5}\text{7}}$ to obtain coset-graph cycles for the construction of an S-box with high nonlinearity. Ali et al. [12] have exploited the dispersion property and the PWLCM map to design S-boxes with improved nonlinearity and low differential probabilities. Arshad et al. [18] applied the concept of Mobius group actions and Galois fields to design the graph-based S-boxes with good cryptographic characteristics. Based on a 3D chaotic map, Liu et al. [13] proposed a dynamic keyed S-box with good performance in color image encryption. Arshad [19] presented lightweight S-box using elliptic curve point and algebraic groups which can be used for steganographic application. Razaq et al. [20] designed a reliable S-box for secure communication using parametrized coset graphs and a new row and column matrix operation. Rashid et al. combined cryptography, steganography, and machine learning for secure Internet data transmission [29]. Tita et al. [21] studied the construction of S-boxes from irreducible polynomial over $GF({\text{2}}^{\text{8}})$ which minimizes correlation and maximizes nonlinearity. Artuger [30] used Josephus logic in the design of three S-boxes that have improved cryptographic performance. Ullah et al. [14] introduced the fractional order Rabinovich-Fabrikant system with key based permutation for generating strong S-boxes. Shadab et al. [25] proposed HawkBoost, hybrid HHO-Booster algorithm that can be used for constructing highly nonlinear S-boxes. Jawed and Sajid [28] suggested COBLAH, a hybrid chaotic-algorithmic heuristic framework capable of outperforming more than twenty benchmark S-box designs. A bio-inspired encryption method based on DNA and RNA sequence transformations was proposed in [31]. Alali et al. [32] designed S-boxes based on structural features for graphs to increase the security of facial recognition and cipher systems. Song et al. [33] developed S-boxes based on unbalanced Feistel structures to achieve better security and lower hardware complexity. Jia et al. [26] employed greedy algorithms and SAT-based search techniques in order to design 4-bit S-boxes with minimal area and optimal resistance to cryptanalysis. Tolpa et al. [15] have improved sine, logistic and tent maps to design lightweight S-boxes with better SAC, BIC and encryption strength. In [34], the authors distributed encrypted text across multiple cover images using a randomized codebook. Qu et al. introduced a heterogeneous coupled-neuron model for medical image encryption, showing that nonlinear dynamical structures remain useful for improving image confidentiality [35]. Recent image-encryption research has also explored high-dimensional discrete hyperchaotic maps with hardware-oriented implementations to improve the security of visual data [36]. Dynamic cryptographic transformations have also been investigated for secure data transmission in Medical IoT, including a dynamic Hill-cipher framework designed to strengthen encryption in resource-connected healthcare environments [37].

Taken together, the reviewed studies show that strong S-boxes can be obtained through chaotic, algebraic, heuristic, and hybrid methods. However, a recurring separation remains between mathematical transparency and systematic numerical refinement. Chaos-based and heuristic methods can improve selected cryptographic scores, but the resulting search path may depend on numerical parameters or stochastic choices. Group-theoretic methods provide a reproducible algebraic starting point, but the initial structure does not necessarily produce the strongest possible nonlinearity and differential properties without further refinement. This motivates a two-stage construction in which an algebraically defined initial S-box is followed by an exhaustive search over a finite and explicitly described permutation group.

### 1.3. Research gaps and problem statement

The explosive development of multimedia applications, social networks, cloud storage, telemedicine and surveillance systems has resulted in an unprecedented volume of digital images being generated, transmitted and stored across public networks. Images have high redundancy, strong pixel correlation and large data size unlike textual information, and so conventional text-oriented ciphers are not very efficient and secure when applied without adaptation. This issue becomes more critical for sensitive visual data, where unauthorized access, visual inference, or privacy leakage may cause serious security concerns [38]. This has motivated the development of specialized image encryption schemes that can efficiently decorrelate pixels, provide strong confusion and diffusion, and resist a wide range of cryptanalytic, statistical, and differential attacks. In most image encryption architectures, the substitution box (S-box) plays a central role as the primary nonlinear component responsible for confusion. A well-designed S-box contributes significantly to breaking statistical regularities in image histograms, reducing pixel correlations, and enhancing resistance against linear and differential cryptanalysis. Thus, it is essential to design S-boxes with a high level of nonlinearity, low differential uniformity, favorable avalanche effect and high algebraic complexity to ensure secure image encryption schemes.

While many S-box construction techniques have been published, there are still some important issues that are not addressed when these S-boxes are used for secure image encryption. The first challenge is achieving a balanced cryptographic profile. Some S-boxes exhibit good nonlinearity but moderate differential uniformity, high linear approximation probability, or non-ideal avalanche behavior. This imbalance is crucial for image encryption, as image data are highly redundant and have strong local pixel correlation, and any minor deficiency in the confusion or diffusion process can leave statistical traces in the encrypted image.

Another challenge is that many of the existing designs do not have a clearly structured refinement mechanism. There are several methods to enhance S-boxes by random permutation, heuristic search, or matrix rearrangement. While these techniques can help improve some numerical properties, they do not necessarily give a clear algebraic path from the original construction to the final S-box. Hence, a systematic, reproducible, and mathematically sound refinement process is needed that is sufficiently large to investigate a rich class of candidate S-boxes.

Prior group-based S-box constructions have used other triangle groups or coset-graph techniques. For instance, Razaq et al. used Δ(2,3,8) over GF(257) to build an S-box [17], and earlier work used Δ(2,3,4) with symmetric-group permutations [16]. The work in [20] used two coset graphs and a matrix row/column shuffle, achieving an average non-linearity 107.75. None of these prior studies used Δ(2,3,43) or our specific refinement group. In contrast, our work employs Δ(2,3,43) and then refines the initial S-box via the group $C_{\text{7406532}}\times C_{\text{3}}$. This is not a simple random positional permutation; rather, it is a structured refinement based on the action of the specifically defined group $C_{\text{7}\text{4}\text{0}\text{6}\text{5}\text{3}\text{2}}\times C_{\text{3}}$, whose 22,219,596 elements are tested on the Δ(2,3,43)−based initial S-box. As a result, the average nonlinearity is raised from 106 to 112 in our design. These choices and outcomes distinguish this paper from the authors’ earlier S-box designs.

To position the proposed framework against the closest group-theoretic S-box constructions, Table 2 compares their algebraic foundations, initial construction procedures, refinement mechanisms, search characteristics, and reported cryptographic performance.

**Table 2:** Comparison of closely related group-theoretic S-box methods.

Method	Algebraic basis	Initial construction	Refinement strategy	Search character	Reported average NL	DU
Coset-graph symmetric-permutation method [16]	Δ(2,3,4) and coset graphs	Values derived from parametrised coset-graph structure	Symmetric-group permutations	Structured permutation family	112	4
Cycle-based triangle-group method [17]	Δ(2,3,8) over $F_{\text{2}\text{5}\text{7}}$	Cycle-based group-theoretic construction	Group-based rearrangement	Algebraically generated candidates	113.75	10
Dual coset-graph matrix method [20]	Pair of parametrised coset graphs	Two graph-derived sequences	Row and column matrix operations	Prescribed matrix rearrangement	107.75	10
Proposed two-stage group-action method	Δ(2,3,43) over $F_{\text{2}\text{5}\text{7}}$	Six equal 43-cycles; all 6! orderings tested	Exhaustive action of $C_{\text{7}\text{4}\text{0}\text{6}\text{5}\text{3}\text{2}}\times C_{\text{3}}$	22,219,596 candidates	112	4

The main contributions of this work are summarized as follows:

- We suggest a novel framework of S-box construction using a parameterized permutation representation of the triangle group $\Delta (\text{2},\text{3},\text{4}\text{3})=\left\langle \gamma ,\tau |\gamma^{\text{2}}=\tau^{\text{3}}=(\gamma \tau {)}^{\text{4}\text{3}}=\text{1}\right\rangle$ on the projective line over $F_{\text{2}\text{5}\text{7}}$.
- We apply a structured refinement method based on the action of the permutation group $C_{\text{7406532}}\times C_{\text{3}}$ on the initial S-box. This structured transformation improves the cryptographic profile of the S-box, most notably raising its nonlinearity from 106 to 112.
- We provide an extensive cryptographic analysis of the proposed S-box showing its excellent performance in several ways such as nonlinearity (avg. 112), bit independence criterion, strict avalanche criterion, differential uniformity, and linear approximation probability.
- A comparative study with many of the recent S-box designs confirms that our proposed S-box provides a balanced and competitive security profile, especially in terms of nonlinearity.
- To demonstrate its practical utility, we develop a strong multi-round image encryption scheme that incorporates the proposed S-box in every encryption round. Entropy, correlation, chi-square, NPCR, UACI, and PSNR analyses demonstrate strong and competitive statistical performance, with the proposed scheme achieving high NPCR values for all three test images.

The remainder of the paper is organised as follows. Section 2 introduces the group-theoretic and finite-field preliminaries required for the construction. Section 3 presents the two-stage S-box design, including the Δ(2,3,43)-based initial construction and the exhaustive group-action refinement. Section 4 evaluates the final S-box using nonlinearity, bit-independence, avalanche, differential, and linear criteria. Section 5 describes the image-encryption and decryption procedures, experimental results, robustness tests, and computational cost. Section 6 summarises the main findings, limitations, and directions for future work.

## 2. Algebraic preliminaries

This section outlines the group-theoretic background underlying the proposed S-box construction. Our framework is based on a parameterized permutation representation of the triangle group Δ(2,3,n) on the projective line over finite fields and the induced cycle structure of its generators.

### 2.1. Permutation representation on $F_{p}\cup \left\{\infty \right\}$

Consider two Möbius transformations $(z)\gamma =\frac{-\text{1}}{z}$ and$(z)\tau =\frac{z-\text{1}}{z}$ satisfying $\gamma^{\text{2}}=\text{1}$ and $\tau^{\text{3}}=\text{1}$, acting on $F_{p}\cup \left\{\infty \right\}$. Applying these generators to each field element yields permutation representations of γ and τ. Since γ has order two, its action decomposes into disjoint transpositions, while τ, being of order three, decomposes into disjoint 3-cycles.

The permutation representation is completely described by the cycle decompositions of γ and τ. In particular, the action of γ yields disjoint transpositions, while the action of τ yields disjoint 3-cycles. These cycle structures provide the algebraic information needed for the subsequent S-box construction.

In the next example, we compute the permutation representation generated by γ and τ on $F_{\text{1}\text{9}}\cup \left\{\infty \right\}$.

**Example 2.1.** We apply the maps $(z)\gamma =\frac{-\text{1}}{z}$ and$(z)\tau =\frac{z-\text{1}}{z}$ to each element of $F_{\text{1}\text{9}}\cup \left\{\infty \right\}$. For every ${z\in F}_{\text{1}\text{9}}\cup \left\{\infty \right\}$, the images (z)γ and (z)τ appear in fractional form. Because arithmetic is performed modulo 19, we repeatedly add 19 to the numerator until the expression reduces to an integer in the field. This procedure yields the following permutation representations of γ and τ:


$$\begin{array}{c} \gamma =(\text{0},\infty )(\text{1},\text{1}\text{8})(\text{2},\text{9})(\text{3},\text{6})(\text{4},\text{1}\text{4})(\text{5},\text{1}\text{5})(\text{7},\text{8})(\text{1}\text{0},\text{1}\text{7})(\text{1}\text{1},\text{1}\text{2})(\text{1}\text{3},\text{1}\text{6}) \end{array}$$

$$\begin{array}{c} \tau =(\text{0},\infty ,\text{1})(\text{2},\text{1}\text{0},\text{1}\text{8})(\text{3},\text{7},\text{9})(\text{4},\text{1}\text{5},\text{6})(\text{5},\text{1}\text{6},\text{1}\text{4})(\text{1}\text{1},\text{1}\text{3},\text{1}\text{7})(\text{8})(\text{1}\text{2}). \end{array}$$

These permutations illustrate the induced action of the generators on $F_{\text{1}\text{9}}\cup \{\infty \}$.

### 2.2. Parameterized permutation representations via Mushtaq’s method

The permutation representation in the above example corresponds to the group $\left\langle \gamma ,\tau |\gamma^{\text{2}}=\tau^{\text{3}}=(\gamma \tau {)}^{\text{1}\text{9}}=\text{1}\right\rangle$. In particular, the relation $(\gamma \tau {)}^{\text{1}\text{9}}=\text{1}$ holds on the projective line over $F_{\text{1}\text{9}}$, confirming the required order of the product γτ.

Mushtaq demonstrated in [39] that a parameterized family of permutation representations can be constructed over $F_{p}$. A key feature of this approach is that it allows the order n of the product γτ to be fixed in advance, making it possible to realize permutation representations associated with triangle groups $\left\langle \gamma ,\tau |\gamma^{\text{2}}=\tau^{\text{3}}=(\gamma \tau {)}^{n}=\text{1}\right\rangle$.

The parametrization method can be outlined as follows (see [39] for a complete treatment). The construction begins with two Möbius transformations $(z)\gamma =\frac{az+kc}{cz-a},(z)\tau =\frac{dz+kf}{fz-d-\text{1}}$ acting on $F_{p}\cup \left\{\infty \right\}$. The constants $a,c,d,k,f\in F_{p}$ are not chosen arbitrarily. Instead, they are determined by a parameter $\theta \in F_{p}$ that controls the order of the product γτ.

Suppose we wish to construct a permutation representation in which $(\gamma \tau {)}^{n}=\text{1}$ on the underlying projective line. To achieve this, the parameter 𝜃 must be a root of a specific polynomial over $F_{p}$. Mushtaq defined this polynomial as


$$\begin{array}{c} f_{n}(\theta )=\left\{ \begin{array}{cc} \;\;\;\;\;\left(\begin{array}{c} @ln-\text{1}\\ \text{0} \end{array}\right)\theta^{\frac{n-\text{1}}{\text{2}}}-\left(\begin{array}{c} @ln-\text{2}\\ \text{1} \end{array}\right)\theta^{\frac{n-\text{3}}{\text{2}}}+\left(\begin{array}{c} @ln-\text{3}\\ \text{2} \end{array}\right)\theta^{\frac{n-\text{5}}{\text{2}}}-\ldots , & \text{if }n\text{ is odd}\\ \left(\begin{array}{c} @ln-\text{1}\\ \text{0} \end{array}\right)\theta^{\frac{n}{\text{2}}-\text{1}}-\left(\begin{array}{c} @ln-\text{2}\\ \text{1} \end{array}\right)\theta^{\frac{n}{\text{2}}-\text{2}}+\left(\begin{array}{c} @ln-\text{3}\\ \text{2} \end{array}\right)\theta^{\frac{n}{\text{2}}-\text{3}}-\ldots ,\; & \text{if }n\text{ is even} \end{array}\right. \end{array}$$

Each root $\theta \in F_{p}$ yields one complete parametrization, and hence one permutation representation.

After selecting such a value of θ, the parameters a,c,d,k,f are computed from the system of equations


$$\begin{array}{c} \theta =\frac{r^{\text{2}}}{\Delta } \end{array}$$ (2.1)

$$\begin{array}{c} r^{\text{2}}+ks^{\text{2}}=\text{3}\Delta \end{array}$$ (2.2)

$$\begin{array}{c} d^{\text{2}}+d+kf^{\text{2}}+\text{1}=\text{0} \end{array}$$ (2.3)

$$\begin{array}{c} a(\text{2}d+\text{1})+\text{2}kcf-r=\text{0} \end{array}$$ (2.4)

$$\begin{array}{c} \text{2}af-c(\text{2}d+\text{1})-s=\text{0} \end{array}$$ (2.5)

equations (2.1)–(2.5) ensure that the transformations $(z)\gamma =\frac{az+kc}{cz-a}$ and $(z)\tau =\frac{dz+kf}{fz-d-\text{1}}$ satisfy


$$\begin{array}{c} \gamma^{\text{2}}=\text{1},\tau^{\text{3}}=\text{1},(\gamma \tau {)}^{n}=\text{1}. \end{array}$$

Once the parameters are determined, the induced permutations of γ and τ on $F_{p}\cup \left\{\infty \right\}$ are computed. These permutations contain only 2-cycles for 𝛾 and 3-cycles for τ. Different choices of θ yield distinct permutation representations and provide a full family of algebraically structured actions over $F_{p}$.

## 3. S-box construction

A parameterized permutation representation and a permutation group serve as the foundation for the suggested S-box design scheme. This section outlines the procedure they employ to complete the work.

### 3.1. Permutation representation employed in the proposed scheme

The construction of the proposed S-box is based on a parameterized permutation representation of the triangle group $\left\langle \gamma ,\tau |\gamma^{\text{2}}=\tau^{\text{3}}=(\gamma \tau {)}^{\text{4}\text{3}}=\text{1}\right\rangle$ acting on $F_{\text{2}\text{5}\text{7}}\cup \{\infty \}$. The choice of $F_{\text{2}\text{5}\text{7}}$ is deliberate, as an 8×8 S-box requires 256 distinct values. The group action is possible on the $F_{p}\cup \{\infty \}$, where p is a prime, and 257 is the nearest prime to 256.

The selection n=43 is connected directly to the size of the projective line. Since $\left|P^{\text{1}}\left(F_{\text{2}\text{5}\text{7}}\right)\right|=\text{2}\text{5}\text{7}+\text{1}=\text{2}\text{5}\text{8}=\text{6}\times \text{4}\text{3},$ an admissible representation in which γτ has order 43 can partition the projective line into six equal cycles of length 43. These six cycles provide the algebraic blocks used in Stage 1. Removing the two auxiliary symbols 256 and ∞ leaves exactly the 256 values required for an 8×8 S-box. The six cycles can also be ordered in 6!=720 ways, giving a finite and fully enumerable family of initial candidates. Thus, n=43 was chosen because it is arithmetically compatible with $\left|P^{\text{1}}\left(F_{\text{2}\text{5}\text{7}}\right)\right|$ and produces a useful equal-cycle decomposition. We do not claim that n=43 is globally optimal over all triangle groups; rather, the present construction is one principled instance of the wider parametric family Δ(2,3,n).

Following Mushtaq’s framework, the construction begins by selecting a parameter θ that ensures the product γτ has order 43. As shown in [39], this requirement forces θ to be a root of the polynomial


$$\begin{array}{cc} f(\theta )= & \theta^{\text{2}\text{1}}-\text{4}\text{1}\theta^{\text{2}\text{0}}+\text{7}\text{8}\text{0}\theta^{\text{1}\text{9}}-\text{9}\text{1}\text{3}\text{9}\theta^{\text{1}\text{8}}+\text{7}\text{3}\text{8}\text{1}\text{5}\theta^{\text{1}\text{7}}-\text{4}\text{3}\text{5}\text{8}\text{9}\text{7}\theta^{\text{1}\text{6}}+\text{1}\text{9}\text{4}\text{7}\text{7}\text{9}\text{2}\theta^{\text{1}\text{5}}\\ & \;\;\;\;-\text{6}\text{7}\text{2}\text{4}\text{5}\text{2}\text{0}\theta^{\text{1}\text{4}}+\text{1}\text{8}\text{1}\text{5}\text{6}\text{2}\text{0}\text{4}\theta^{\text{1}\text{3}}-\text{3}\text{8}\text{5}\text{6}\text{7}\text{1}\text{0}\text{0}\theta^{\text{1}\text{2}}+\text{6}\text{4}\text{5}\text{1}\text{2}\text{2}\text{4}\text{0}\theta^{\text{1}\text{1}}\\ & \;\;\;\;-\text{8}\text{4}\text{6}\text{7}\text{2}\text{3}\text{1}\text{5}\theta^{\text{1}\text{0}}+\text{8}\text{6}\text{4}\text{9}\text{3}\text{2}\text{2}\text{5}\theta^{\text{9}}-\text{6}\text{7}\text{8}\text{6}\text{3}\text{9}\text{1}\text{5}\theta^{\text{8}}+\text{4}\text{0}\text{1}\text{1}\text{6}\text{6}\text{0}\text{0}\theta^{\text{7}}\\ & \;\;\;\;-\text{1}\text{7}\text{3}\text{8}\text{3}\text{8}\text{6}\text{0}\theta^{\text{6}}+\text{5}\text{3}\text{1}\text{1}\text{7}\text{3}\text{5}\theta^{\text{5}}-\text{1}\text{0}\text{8}\text{1}\text{5}\text{7}\text{5}\theta^{\text{4}}+\text{1}\text{3}\text{4}\text{5}\text{9}\text{6}\theta^{\text{3}}-\text{8}\text{8}\text{5}\text{5}\theta^{\text{2}}+\text{2}\text{3}\text{1}\theta -\text{1} \end{array}$$

Over $F_{\text{2}\text{5}\text{7}}$, this polynomial has the root θ=176, and this value is used for the remainder of the construction.

To determine the Möbius transformations $(z)\gamma =\frac{az+kc}{cz-a},(z)\tau =\frac{dz+kf}{fz-d-\text{1}}$, we substitute θ=176 into the system of equations (2.1)–(2.5). These equations enforce the relations $\gamma^{\text{2}}=\tau^{\text{3}}=(\gamma \tau {)}^{\text{4}\text{3}}=\text{1}$. Several variables may be chosen freely, and we adopt the following convenient values:

- Set Δ=136. From (2.1) this gives r=99.
- Take k=221. Substituting k and r into (2.2) yields s=232.
- Take d=106. Substituting d and k into (2.3) gives f=145.

Substituting the values r,k,d,s,f into the remaining equations (2.4) and (2.5) and solving for the remaining parameters leads to a=33, c=220.

These “freely chosen” parameters are not unconstrained: each admissible tuple (Δ,k,d) satisfying (2.1)–(2.5) defines specific Möbius maps (z)γ and (z)τ, and thus a particular cycle decomposition of γτ. Different admissible choices can yield different initial S-boxes (even before refinement), so their cryptographic properties may vary. In our construction, the values Δ=136,k=221,d=106 (leading to r=99,s=232,f=145 and then a=33,c=220) were chosen because they satisfy the equations and produce an initial S-box that can be effectively refined. However, one could experiment with other admissible parameters to see how the initial S-box’s strength changes.

Substituting all parameters back gives the explicit forms of the transformations:


$$\begin{array}{c} (z)\gamma =\frac{\text{3}\text{3}z+\text{4}\text{7}}{\text{2}\text{2}\text{0}z+\text{2}\text{2}\text{4}}\text{ and }(z)\tau =\frac{\text{1}\text{0}\text{6}z+\text{1}\text{7}\text{7}}{\text{1}\text{4}\text{5}z+\text{1}\text{5}\text{0}}. \end{array}$$

To compute the required permutation representation, we apply γ and τ to every element of $F_{\text{2}\text{5}\text{7}}\cup \{\infty \}$.For example,


$$\begin{array}{c} (\text{0})\gamma =\frac{\text{4}\text{7}}{\text{2}\text{2}\text{4}}=\text{2}\text{4}\text{0},(\text{1})\gamma =\frac{\text{8}\text{0}}{\text{4}\text{4}\text{4}}=\text{1}\text{0}\text{9}\text{.} \end{array}$$

Carrying out this process for all elements produces the permutation actions:

γ *=(0,240)(1,109)(2,71)(3,231)(4,141)(5,157)(6,251)(7,205)(8,192)(9,44)(10,140)(11,75)(12,137)(13,∞)(14,146)(15,208)(16,143)(17,239)(18,91)(19,78)(20,32)(21,126)(22,142)(23,52)(24,212)(25,174)(26,43)(27,151)(28,39)(29,198)(30,172)(31,206)(33,161)(34,105)(35,241)(36,97)(37,222)(38,80)(40,56)(41,82)(42,248)(45,234)(46,165)(47,221)(48,171)(49,238)(50,93)(51,145)(53,87)(54,104)(55,59)(57,127)(58,193)(60,158)(61,246)(62,236)(63,175)(64,66)(65,149)(67,163)(68,207)(69,176)(70,101)(72,159)(73,148)(74,150)(76,215)(77,252)(79,89)(81,117)(83,92)(84,185)(85,254)(86,131)(88,121)(90,225)(94,113)(95,187)(96,188)(98,199)(99,155)(100,177)(102,130)(103)(106,183)(107,214)(108,220)(110,123)(111,253)(112,235)(114,139)(115,168)(116,164)(118,237)(119,167)(120,216) (122,250)(124,211)(125,223)*

τ *=(0,68,25)(1,163,60)(2,26,227)(3,159,114)(4,211,17)(5,118,90)(6,59,140)(7,93,111)(8,41,188)(9,244,178)(10,203,252)(11,37,246)(12,95,136)(13,134,230)(14,202,219)(15,83,58)(16,210,186)(18,153,120)(19,150,54)(20,197,179)(21,105,40)(22,61,∞)(23,177,82)(24,77,200)(27,165,168)(28,255,193)(29,190,64)(30,212,160)(31,47,149)(32,39,243)(33,214,133)(34,164,127)(35,209,201)(36,52,191)(38,144,239)(42,75,152)(43,235,62)(44,51,97)(45,101,196)(46,72,94)(48,139,131)(49,213,176)(50,207,126)(53,180,128)(55,147,85)(56,172,175)(57,81,113)(63,161,143)(65,220,187)(66,129,79)(67,154,130)(69,121,138)(70,110,206)(71,204,245)(73,88,137)(74,162,96)(76,229,247)(78,250,222)(80,226,181)(84,157,223)(86,249,234)(87,92,89)(91,254,106)(98,224,112)(99,124,108)(100,240,170)(102,104,148)(103,151,185)(107,218,158)(109,167,141)(115,208,169)(116,242,228)(117,183,256)(119,142,146)(122,233,182)(123,184,135)(125,195,166)(132,225,171)(145,215,174)(155,189,237)(156,217,205)(173,231,199)(192,236,238)(194,198,221)(216,241,232)(248,253,251)*

The above permutation representations fully determine the action of γ and τ on $F_{\text{2}\text{5}\text{7}}\cup \{\infty \}$. For the purposes of S-box construction, the key object is the cycle decomposition of the product γτ.

To confirm that the product γτ has order 43, we track the orbit of any element. Starting from 0:


$$\begin{array}{c} (\text{0})\gamma =\text{2}\text{4}\text{0},(\text{2}\text{4}\text{0})\tau =\text{1}\text{7}\text{0}\text{.} \end{array}$$

This shows (0)(γτ)=170. Continuing this process, the orbit eventually returns to 0 after exactly 43 steps. The same behaviour is observed for every element of $F_{\text{2}\text{5}\text{7}}\cup \{\infty \}$, which means the relation $(\gamma \tau {)}^{\text{4}\text{3}}=\text{1}$ is satisfied for all elements of $F_{\text{2}\text{5}\text{7}}\cup \{\infty \}$. So, the permutation γτ is the product of 6 disjoint cycles $\delta_{\text{1}},\delta_{\text{2}}{,\delta }_{\text{3}},\delta_{\text{4}},\delta_{\text{5}}{,\delta }_{\text{6}}$ each of length 43,where

$\delta_{\text{1}}$ *=(0,170,237,90,171,139,3,199,224,116,127,81,183,91,153,80,144,115,27,185,157,118,155,124,17,38,226,217,14,119,141,211,108,187,136,79,87,180,128,135,186,76,174)*

$\delta_{\text{2}}$ *=(1,167,142,61,11,152,179,247,16,63,56,21,50,111,251,59,147,130,104,19,250,233,64,129,12,73,102,67,60,107,133,201,228,112,62,238,213,122,222,246,∞,134,158)*

$\delta_{\text{3}}$ *=(2,204,198,190,182,176,121,137,95,65,31,70,196,13,22,146,202,125,84,103,151,165,72,114,131,249,9,51,215,229,20,39,255,178,234,101,110,184,53,92,58,28,243)*

$\delta_{\text{4}}$ *=(4,109,163,154,85,106,256,225,5,223,195,96,8,236,43,227,32,197,42,253,7,156,181,120,241,209,33,143,210,123,206,47,194,245,252,200,36,44,244,193,15,169,239)*

$\delta_{\text{5}}$ *=(6,248,75,37,78,150,162,166,219,205,93,207,25,145,97,52,177,240,68,126,105,164,242,35,232,69,49,192,41,23,191,24,160,231,159,94,57,34,40,172,212,77,10)*

$\delta_{\text{6}}$ *=(18,254,55,140,203,71,26,235,98,173,30,175,161,214,218,230,45,86,48,132,117,113,46,168,208,83,89,66,29,221,149,220,99,189,100,82,188,74,54,148,88,138,216)*

### 3.2. Proposed S-box method

The proposed approach of S-box construction has following two steps.

***Step 1:*** *Initial construction*

In this step, we use the six 43-cycles $\delta_{\text{1},}\delta_{\text{2},}\delta_{\text{3},}\delta_{\text{4}}\delta_{\text{5},}\delta_{\text{6}}$ of the permutation γτ, obtained from the permutation representation of Δ(2,3,43), to generate the initial S-box. Each of these cycles has 43 elements, that is, we have 6 × 43 = 258 elements in total. We ignore 256and ∞, as they are present only for algebraic reasons and have no role in the S-box construction. There are 6!=720 possible arrangements of the given six cycles, and each such arrangement creates a random sequence of 256 elements ranging from 0 to 255, which can be written in row-major form into a 16 × 16 matrix to obtain an S-box. In this study, we adopt the ordering $\delta_{\text{5}}\delta_{\text{4}}\delta_{\text{1}}\delta_{\text{3}}\delta_{\text{6}}\delta_{\text{2}}$.

This ordering was not chosen randomly. The six cycles can be arranged in 6!=720 ways, so we tested all the cycle orderings and created the initial S-boxes. The ordering $\delta_{\text{5}}\delta_{\text{4}}\delta_{\text{1}}\delta_{\text{3}}\delta_{\text{6}}\delta_{\text{2}}$ was chosen because it yielded the most appropriate initial S-box in terms of average nonlinearity and minimum component Boolean nonlinearity, and hence provided a better starting point for the Stage 2 refinement. The resulting S-box is presented in Table 3.

**Table 3:** Initial S-box.

*6*	*248*	*75*	*37*	*78*	*150*	*162*	*166*	*219*	*205*	*93*	*207*	*25*	*145*	*97*	*52*
*177*	*240*	*68*	*126*	*105*	*164*	*242*	*35*	*232*	*69*	*49*	*192*	*41*	*23*	*191*	*24*
*160*	*231*	*159*	*94*	*57*	*34*	*40*	*172*	*212*	*77*	*10*	*4*	*109*	*163*	*154*	*85*
*106*	*225*	*5*	*223*	*195*	*96*	*8*	*236*	*43*	*227*	*32*	*197*	*42*	*253*	*7*	*156*
*181*	*120*	*241*	*209*	*33*	*143*	*210*	*123*	*206*	*47*	*194*	*245*	*252*	*200*	*36*	*44*
*244*	*193*	*15*	*169*	*239*	*0*	*170*	*237*	*90*	*171*	*139*	*3*	*199*	*224*	*116*	*127*
*81*	*183*	*91*	*153*	*80*	*144*	*115*	*27*	*185*	*157*	*118*	*155*	*124*	*17*	*38*	*226*
*217*	*14*	*119*	*141*	*211*	*108*	*187*	*136*	*79*	*87*	*180*	*128*	*135*	*186*	*76*	*174*
*2*	*204*	*198*	*190*	*182*	*176*	*121*	*137*	*95*	*65*	*31*	*70*	*196*	*13*	*22*	*146*
*202*	*125*	*84*	*103*	*151*	*165*	*72*	*114*	*131*	*249*	*9*	*51*	*215*	*229*	*20*	*39*
*255*	*178*	*234*	*101*	*110*	*184*	*53*	*92*	*58*	*28*	*243*	*18*	*254*	*55*	*140*	*203*
*71*	*26*	*235*	*98*	*173*	*30*	*175*	*161*	*214*	*218*	*230*	*45*	*86*	*48*	*132*	*117*
*113*	*46*	*168*	*208*	*83*	*89*	*66*	*29*	*221*	*149*	*220*	*99*	*189*	*100*	*82*	*188*
*74*	*54*	*148*	*88*	*138*	*216*	*1*	*167*	*142*	*61*	*11*	*152*	*179*	*247*	*16*	*63*
*56*	*21*	*50*	*111*	*251*	*59*	*147*	*130*	*104*	*19*	*250*	*233*	*64*	*129*	*12*	*73*
*102*	*67*	*60*	*107*	*133*	*201*	*228*	*112*	*62*	*238*	*213*	*122*	*222*	*246*	*134*	*158*

**Step 2.** *Refinement of the initial S-box using group actions*

In this step, we generate the proposed S-box by utilizing the action of the group $G=C_{\text{7406532}}\times C_{\text{3}}$ on the initial S-box. The refinement group was selected to provide a structured search space for improving the initial S-box without destroying its bijective nature. The initial S-box obtained from the Δ(2,3,43)-based construction has average nonlinearity 106. Since the nonlinearity of an S-box depends on the arrangement of its truth-table entries, a suitable positional permutation can improve the nonlinear behavior of its component Boolean functions. Therefore, instead of using random permutations, we used the group $G=C_{\text{7406532}}\times C_{\text{3}}$, whose 22,219,596 elements form a large but computationally manageable search space. Each element acts only on the positions of the initial S-box entries, preserving all 256 distinct output values. By testing all group elements, the best element $a^{\text{7}\text{5}}b^{\text{9}\text{5}}c^{\text{1}\text{1}}d^{\text{2}\text{3}}e^{\text{2}}$ was found to increase the average nonlinearity from 106 to 112.

The refinement group is based on commuting cyclic actions on disjoint position blocks. The generators a,b,c,d,e are cycles of lengths 101,97,27,28, and 3, respectively, and their supports are disjoint. Their support sizes add to 256, so together they act on every position of the flattened S-box. Because permutations with disjoint supports commute, every group element has a unique form


$$\begin{array}{c} a^{i}b^{j}c^{k}d^{l}e^{m}, \end{array}$$

where 0≤i<101, 0≤j<97, 0≤k<27, 0≤l<28, and 0≤m<3. Moreover, 101,97,27, and 28 are pairwise coprime, so


$$\begin{array}{c} C_{\text{1}\text{0}\text{1}}\times C_{\text{9}\text{7}}\times C_{\text{2}\text{7}}\times C_{\text{2}\text{8}}≅C_{\text{1}\text{0}\text{1}\cdot \text{9}\text{7}\cdot \text{2}\text{7}\cdot \text{2}\text{8}}=C_{\text{7}\text{4}\text{0}\text{6}\text{5}\text{3}\text{2}}. \end{array}$$

Hence,


$$\begin{array}{c} G≅C_{\text{7}\text{4}\text{0}\text{6}\text{5}\text{3}\text{2}}\times C_{\text{3}},\;\;|G|=\text{2}\text{2},\text{2}\text{1}\text{9},\text{5}\text{9}\text{6}. \end{array}$$

This construction provides a large but exactly enumerable set of position permutations. Every candidate remains bijective because the group changes only the positions of the 256 distinct output values. The selected cycle partition is not claimed to be canonical or globally optimal; it was designed to give full positional coverage, commuting generators, a unique exponent representation, and an exhaustive search size that remains computationally practical.

The group G is generated by five permutations a,b,c,d and e acting on {1,2,3,…,256}. Their cycle decompositions are:

*a=(1,96,103,24,221,133,136,42,25,113,12,40,248,119,26,112,159,18,222,5,255,124,90,150,154,91,126,157,80,237,243,176,98,215,97,54,192,58,106,63,183,137,204,228,45,48,99,60,234,166,95,217,15,72,230,211,110,20,9,172,238,2,4,85,162,53,43,220,65,116,8,208,73,51,57,165,214,66,37,94,49,77,171,190,109,21,107,125,245,209,152,242,142,121,182,75,201,38,132,29,224)*

*b=(3,86,218,114,88,105,170,62,120,19,184,253,129,179,194,44,198,27,181,193,177,35,87,70,205,186,226,30,46,128,145,207,191,23,175,111,144,64,140,247,196,134,102,76,229,71,22,47,41,212,50,163,69,180,189,52,36,199,115,200,11,6,246,195,135,92,131,93,158,161,155,241,7,169,68,151,256,188,28,32,55,149,82,185,164,225,227,108,138,236,118,216,139,14,160,100,146)*

*c=(10,206,33,219,187,240,84,231,178,143,197,17,39,250,213,233,249,203,254,61,202,101,13,79,16,31,74)*

*d=(34,81,89,59,122,117,244,252,123,147,83,174,130,173,210,167,168,239,104,232,156,251,148,141,223,67,56,153)*

*e = (78,235,127)*

The GAP software shows that $C_{\text{7406532}}\times C_{\text{3}}$ has the presentation:


$$\begin{array}{cc} & \langle a,b,c,d,e:a^{\text{1}\text{0}\text{1}}=b^{\text{9}\text{7}}{=c}^{\text{2}\text{7}}{=d}^{\text{2}\text{8}}{=e}^{\text{3}}=aba^{-\text{1}}b^{-\text{1}}=aca^{-\text{1}}c^{-\text{1}}=ada^{-\text{1}}d^{-\text{1}}\\ & =aea^{-\text{1}}e^{-\text{1}}=bcb^{-\text{1}}c^{-\text{1}}=bdb^{-\text{1}}d^{-\text{1}}=beb^{-\text{1}}e^{-\text{1}}=cdc^{-\text{1}}d^{-\text{1}}=cec^{-\text{1}}e^{-\text{1}}=ded^{-\text{1}}e^{-\text{1}}=\text{1}\rangle , \end{array}$$

and that the group is of order 22,219,596.

Note that the refinement group$C_{\text{7406532}}\times C_{\text{3}}$ is specifically constructed for the Δ(2,3,43)-based S-box and has no counterpart in the earlier works. Unlike previous schemes that applied random permutations or different matrix operations, here we exhaustively search this direct-product group to improve nonlinearity. This refinement not only enhances the average nonlinearity from 106 to 112, but also improves the overall cryptographic profile of the S-box, such as BIC, DU, and LAP values, while maintaining SAC close to its ideal value. These improvements can also be seen from Table 10, in which the refined S-box has better and more balanced performance than the initial S-box.

We denote the initial S-box by $S_{\text{0}}$ and flatten it into a vector:


$$\begin{array}{c} S_{\text{0}}=\left\{s_{\text{0}},s_{\text{1}},s_{\text{2}},\ldots ,s_{\text{2}\text{5}\text{5}}\right\}. \end{array}$$

For any permutation g∈G, the action is defined as:


$$\begin{array}{c} S_{g}[i]=S_{\text{0}}\left[g^{-\text{1}}(i)\right]. \end{array}$$

The above equation shows that the entry of the initial S-box lying at position $g^{-\text{1}}(i)$ is moved to the *ith* position of the proposed S-box. The result of this operation is a pure positional rearrangement which leaves the 256 different output values intact. The elements of G act on the position of the entries rather than the entries themselves. The group G has 22,219,596 elements, and all of them were applied to the initial S-box. Each resulting S-box was evaluated for component nonlinearity using its Walsh spectra. The candidate with the highest average component nonlinearity was selected, after which the final S-box was evaluated using SAC, BIC, differential uniformity, and linear approximation probability. The best S-box was obtained when the element $a^{\text{7}\text{5}}b^{\text{9}\text{5}}c^{\text{1}\text{1}}d^{\text{2}\text{3}}e^{\text{2}}$ acts on $S_{\text{0}}.$ We call it our proposed S-box, and it is presented in Table 4. The complete exhaustive Stage 2 implementation is publicly available at https://github.com/abdulrazaq-ship-it/Algebraic-SBox-Exhaustive-Refinement. A step-by-step positional illustration of how this group element rearranges the entries of the initial S-box is provided in S1 Appendix.

**Table 4:** Final S-box.

*186*	*135*	*61*	*133*	*85*	*168*	*209*	*221*	*124*	*62*	*201*	*117*	*177*	*153*	*57*	*238*
*205*	*111*	*222*	*48*	*207*	*212*	*38*	*64*	*81*	*175*	*113*	*8*	*249*	*174*	*138*	*151*
*246*	*211*	*143*	*119*	*35*	*171*	*100*	*227*	*225*	*1*	*13*	*49*	*166*	*202*	*88*	*188*
*182*	*33*	*41*	*66*	*67*	*248*	*193*	*32*	*63*	*239*	*180*	*5*	*26*	*68*	*195*	*228*
*30*	*115*	*90*	*158*	*86*	*218*	*154*	*224*	*190*	*104*	*134*	*210*	*137*	*250*	*40*	*59*
*107*	*101*	*53*	*25*	*74*	*14*	*189*	*28*	*122*	*19*	*142*	*199*	*255*	*179*	*216*	*215*
*129*	*219*	*206*	*75*	*83*	*251*	*44*	*196*	*253*	*178*	*172*	*233*	*217*	*243*	*156*	*95*
*96*	*185*	*93*	*194*	*15*	*31*	*7*	*161*	*247*	*84*	*254*	*197*	*112*	*97*	*200*	*82*
*46*	*12*	*229*	*165*	*203*	*245*	*198*	*183*	*152*	*108*	*39*	*208*	*231*	*240*	*191*	*70*
*132*	*0*	*54*	*131*	*214*	*184*	*45*	*226*	*87*	*116*	*162*	*241*	*123*	*9*	*99*	*125*
*102*	*114*	*98*	*50*	*6*	*110*	*51*	*213*	*72*	*136*	*77*	*105*	*27*	*92*	*146*	*126*
*170*	*52*	*4*	*223*	*71*	*78*	*10*	*2*	*56*	*23*	*149*	*24*	*94*	*232*	*140*	*37*
*159*	*89*	*3*	*144*	*47*	*173*	*29*	*150*	*128*	*22*	*169*	*181*	*21*	*220*	*242*	*34*
*69*	*130*	*252*	*234*	*11*	*127*	*18*	*145*	*120*	*237*	*42*	*79*	*60*	*109*	*244*	*139*
*155*	*163*	*65*	*141*	*164*	*106*	*36*	*16*	*230*	*43*	*76*	*167*	*148*	*118*	*103*	*80*
*58*	*20*	*17*	*55*	*187*	*121*	*176*	*157*	*73*	*160*	*236*	*204*	*235*	*147*	*91*	*192*

As an additional structural check, we examined both the initial and the final S-box as permutations on the set {0,1,…,255}. The refined S-box has no fixed points, that is, there is no x such that S(x)=x, and it also has no reverse fixed points, that is, there is no x such that S(x)=255−x. Its cycle decomposition consists of three disjoint cycles of lengths 50,98, and 108. Hence, the proposed S-box contains no 1-cycles, 2-cycles, or other short periodic cycles. The initial S-box also has no fixed points, but it contains two reverse fixed points, namely x=119 and x=134. Therefore, the refinement stage not only improves the standard cryptographic metrics, but also removes reverse fixed points and yields a cleaner cycle structure.

## 4. Assessment of performance and comparative analysis

This section presents a detailed assessment of the proposed 8×8 S-box. A secure S-box must satisfy several well-established cryptographic properties that ensure a nonlinear and unpredictable relationship between the input and output. These properties also indicate the S-box’s resistance to common forms of cryptanalysis. The evaluation here considers the standard criteria listed below:

- Nonlinearity (NL)
- Bit Independence Criterion (BIC)
- Strict Avalanche Criterion (SAC)
- Differential Uniformity (DU)
- Linear Approximation Probability (LAP)

### 4.1. Nonlinearity

Nonlinearity is one of the most important measures of S-box strength. A highly nonlinear mapping reduces the success of linear cryptanalysis by ensuring that no linear function closely approximates the Boolean functions that make up the S-box.

For a Boolean function g, the nonlinearity is defined as


$$\begin{array}{c} {NL}_{g}={\text{2}}^{n-\text{1}}\left({\text{1}-{\text{2}}^{-n}}_{c\in F_{\text{2}}^{n}}^{max}|K_{g}(c)|\right) \end{array}$$

where n=8 is the number of input bits, and $K_{g}(c)=\sum_{x\in F_{\text{2}}^{n}}{\left(-\text{1}\right)}^{g(x)⊕(c.x)}$ is the Walsh–Hadamard transform. The theoretical maximum nonlinearity for a balanced 8-variable Boolean function is 120. Table 5 presents the nonlinearity values of the eight component functions $\varphi_{\text{1}}$-$\varphi_{\text{8}}$. The average non-linearity score of the proposed S-box is 112, which indicates that it is strongly resistant to linear attacks.

**Table 5:** Nonlinearity values of the eight component Boolean functions.

Boolean function	$\varphi_{1}$	$\varphi_{2}$	$\varphi_{3}$	$\varphi_{4}$	$\varphi_{5}$	$\varphi_{6}$	$\varphi_{7}$	$\varphi_{8}$
Score	*112*	*112*	*112*	*112*	*112*	*112*	*112*	*112*

### 4.2. Bit Independence Criterion (BIC)

The Bit Independence Criterion evaluates whether pairs of output bits change independently when a single input bit is flipped. When a single input bit is flipped, the resulting changes in each pair of output bits should be statistically independent. An S-box with strong bit independence avoids predictable correlations that adversaries could exploit. Tables 6 and 7 present the BIC- nonlinearity values and the BIC-SAC values. These findings indicate that the S-box proposed has good output bit independence and can be used to achieve uniform avalanche behavior between output pairs.

**Table 6:** BIC Nonlinearity values of newly generated S-box.

*–*	*112*	*112*	*112*	*112*	*112*	*112*	*112*
*112*	*–*	*112*	*112*	*112*	*112*	*112*	*112*
*112*	*112*	*–*	*112*	*112*	*112*	*112*	*112*
*112*	*112*	*112*	*–*	*112*	*112*	*112*	*112*
*112*	*112*	*112*	*112*	*–*	*112*	*112*	*112*
*112*	*112*	*112*	*112*	*112*	*–*	*112*	*112*
*112*	*112*	*112*	*112*	*112*	*112*	*–*	*112*
*112*	*112*	*112*	*112*	*112*	*112*	*112*	*–*

**Table 7:** BIC-SAC values for the proposed S-box.

*–*	*0.502*	*0.5117*	*0.4941*	*0.4883*	*0.5039*	*0.502*	*0.5*
*0.502*	*–*	*0.5195*	*0.502*	*0.4863*	*0.5098*	*0.4922*	*0.4922*
*0.5117*	*0.5195*	*–*	*0.4863*	*0.5254*	*0.5117*	*0.5156*	*0.4961*
*0.4941*	*0.502*	*0.4863*	*–*	*0.4609*	*0.4941*	*0.4727*	*0.4941*
*0.4883*	*0.4863*	*0.5254*	*0.4609*	*–*	*0.502*	*0.4863*	*0.498*
*0.5039*	*0.5098*	*0.5117*	*0.4941*	*0.502*	*–*	*0.502*	*0.5078*
*0.502*	*0.4922*	*0.5156*	*0.4727*	*0.4863*	*0.502*	*–*	*0.5215*
*0.5*	*0.4922*	*0.4961*	*0.4941*	*0.498*	*0.5078*	*0.5215*	*–*

For output bits i≠j, the BIC-NL entry is the nonlinearity of the Boolean function $S_{i}(x)⊕S_{j}(x)$.

The uniform entries in Tables 5 and 6 were checked using the complete set of nonzero output masks, rather than only the eight coordinate bits. For each $\beta \in F_{\text{2}}^{\text{8}}\backslash \{\text{0}\}$, we formed the Boolean component


$$\begin{array}{c} f_{\beta }(x)=\beta \cdot S(x) \end{array}$$

and computed its Walsh spectrum. All 255 nonzero components have


$$\begin{array}{c} \underset{\alpha \in F_{\text{2}}^{\text{8}}}{\text{max}}\left|W_{f_{\beta }}(\alpha )\right|=\text{3}\text{2}, \end{array}$$

and hence


$$\begin{array}{c} NL\left(f_{\beta }\right)=\text{1}\text{2}\text{8}-\frac{\text{3}\text{2}}{\text{2}}=\text{1}\text{1}\text{2}. \end{array}$$

The eight coordinate functions in Table 5 correspond to masks of Hamming weight one, while the functions used in Table 6 correspond to masks of Hamming weight two. Their common value of 112 therefore follows from the stronger all-mask result.

### 4.3. Strict Avalanche Criterion (SAC)

A Boolean function satisfies the strict avalanche criterion if any change in a single input bit has a 50% chance of inverting the output bit. Formally, for each unit vector $e_{i}$,


$$\begin{array}{c} \frac{\text{1}}{{\text{2}}^{n}}\sum_{x\in F_{\text{2}}^{n}}h(x)⊕h\left(x⊕e_{i}\right)=\frac{\text{1}}{\text{2}}. \end{array}$$

Table 8 summarizes the SAC results. Most of the entries are close to the optimum value of 0.5, thus, showing that the suggested S-box produces almost perfect avalanche behavior when one input bit is flipped.

**Table 8:** SAC score for the proposed S-box.

*0.4531*	*0.5*	*0.5312*	*0.5156*	*0.5469*	*0.5312*	*0.4844*	*0.5156*
*0.4844*	*0.4531*	*0.4531*	*0.4531*	*0.5*	*0.5469*	*0.5312*	*0.4688*
*0.4531*	*0.5625*	*0.5469*	*0.4531*	*0.4531*	*0.5156*	*0.4531*	*0.5312*
*0.5*	*0.5312*	*0.4531*	*0.5469*	*0.5312*	*0.5312*	*0.5156*	*0.4688*
*0.5156*	*0.4844*	*0.5312*	*0.5312*	*0.5*	*0.5156*	*0.4531*	*0.5469*
*0.5156*	*0.4688*	*0.5156*	*0.5156*	*0.4531*	*0.4531*	*0.5312*	*0.5*
*0.5625*	*0.5*	*0.5*	*0.5312*	*0.5156*	*0.4688*	*0.5156*	*0.5156*
*0.4531*	*0.4844*	*0.5312*	*0.5156*	*0.5*	*0.4688*	*0.4844*	*0.5625*

### 4.4. Differential Uniformity (DU)

Differential uniformity estimates the resistance to differential cryptanalysis. It measures the consistency of the S-box response to pairs of input values that are separated by a constant distance. Smaller values of differential uniformity are a sign of greater resistance to differential attacks. Differential uniformity is defined as


DUf=(maxΔx≠0,Δy)(#{xϵX:f(x)⨁f(x⨁Δx)=Δy})

The differential distribution table is defined by


$$\begin{array}{c} DDT[\Delta x,\Delta y]=\#\{x\in F_{\text{2}}^{\text{8}}:S(x)⊕S(x⊕\Delta x)=\Delta y\}. \end{array}$$

A complete DDT has 256×256 entries. To avoid presenting a large and difficult-to-read matrix in the main text, Table 9 summarises its verified row spectrum.

**Table 9:** Row spectrum of the differential distribution table.

Input-difference category	Number of rows	Number of 0 entries	Number of 2 entries	Number of 4 entries	Other nonzero entries	Row sum
Δx=0	1	255	0	0	One entry equal to 256	256
Δx≠0	255	129 per row	126 per row	1 per row	0	256

For every nonzero input difference, exactly one output difference occurs four times, 126 output differences occur twice, and 129 output differences do not occur. The maximum nontrivial DDT entry is therefore 4, so the proposed S-box is differentially 4-uniform. It is not an almost-perfect nonlinear permutation, because APN mappings have differential uniformity 2. The existence of APN permutations in even dimensions of eight or more remains a well-known open problem; consequently, differential uniformity 4 is an important practical target for 8-bit permutations.

### 4.5. Linear Approximation Probability (LAP)

Linear approximation probability is a measure of the maximum likelihood that a linear relationship between the input and output bits of an S-box exists. It is given by


LAP=(maxα,β≠0)|#{x∈X:α.x=β.f(x)}2n−12|.

In this paper, LAP denotes the maximum absolute linear bias. Let


$$\begin{array}{c} W_{S}(\alpha ,\beta )=\sum_{x\in F_{\text{2}}^{\text{8}}}(-\text{1}{)}^{\alpha \cdot x⊕\beta \cdot S(x)}. \end{array}$$

The verified maximum for nonzero β is


$$\begin{array}{c} \underset{\alpha ,\beta \neq \text{0}}{\text{max}}\left|W_{S}(\alpha ,\beta )\right|=\text{3}\text{2}. \end{array}$$

Therefore, the maximum absolute bias is


$$\begin{array}{c} LAP_{\text{bias}}=\frac{\text{3}\text{2}}{{\text{2}}^{\text{9}}}=\text{0}.\text{0}\text{6}\text{2}\text{5}. \end{array}$$

The corresponding maximum absolute correlation is


$$\begin{array}{c} LAP_{\text{corr}}=\frac{\text{3}\text{2}}{{\text{2}}^{\text{8}}}=\text{0}.\text{1}\text{2}\text{5}. \end{array}$$

This is consistent with the component nonlinearity


$$\begin{array}{c} \text{1}\text{2}\text{8}-\frac{\text{3}\text{2}}{\text{2}}=\text{1}\text{1}\text{2}. \end{array}$$

Thus, the reported value 0.0625 represents the maximum absolute linear **bias,** not the maximum absolute correlation. Both conventions are reported to avoid ambiguity when comparing studies that use different definitions of linear approximation probability.

### 4.6.  Comparison analysis discussion

With the help of group actions and a new refinement procedure, we have created an S-box with an average nonlinearity score of 112, which is very resistant to linear cryptanalysis. Table 10 indicates that although certain contemporary S-boxes might be more advantageous in certain attributes, our design continues to exhibit very good performance in the main cryptographic measures, which gives it good protection against linear and differential attacks.

**Table 10:** Comparison of nonlinearity, bit independence, avalanche criteria, differential uniformity, and linear probability of proposed S-box with existing methods.

S-box	Average NL	BIC-SAC	SAC	BIC-NL	DU	LAP
Proposed S-box	112	0.4996	0.5032	112	4	0.0625
Initial S-box	106	0.5017	0.5017	102.50	16	0.1406
Ali [40]	112	0.5008	0.4988	112	4	0.0625
Mahboob [41]	112.75	0.5010	0.4973	103.64	12	0.13281
Baowidan [42]	112	0.4984	0.5049	112	4	0.0625
Gulraiz [43]	112	0.5039	0.4995	112	4	0.0625
Abbasi [44]	112	0.4984	0.5049	112	4	0.0625
Hazzazi [45]	112	0.4997	0.4971	112	4	0.0625
Ahmed [46]	107.50	0.4982	0.4944	104.35	10	0.1250
Hayat [47]	106.25	0.4975	0.5112	103.93	12	0.1484
Ibrahim [48]	106.5	0.4987	0.5010	103.93	10	0.125
Alshammari [49]	107	0.5019	0.4949	102.29	12	0.141
Alhadawi [50]	108.5	0.5011	0.4995	103.85	10	0.109
Long [51]	109.75	0.4987	0.5042	110.6	6	0.0859
Soto [52]	106.5	0.5019	0.4943	103.35	12	0.1468
Yan [53]	105.5	0.5031	0.5065	103.57	10	0.1328
Zhou [54]	107	0.5050	0.4993	103.29	10	0.1328
Jamal [55]	108	0.5020	0.5029	104.43	14	0.1328
Wang [56]	105	0.5002	0.5063	104.07	10	0.1328
Ejaz [57]	107	0.4985	0.5078	104.43	12	0.1406
Zamli [58]	109.75	0.5041	0.4998	104.14	10	0.1171
Murtaza [59]	106.75	0.4997	0.5051	104.43	10	0.1406
Farah [60]	106.50	0.4983	0.4995	104.57	10	0.1171
Alhadawi [61]	108.5	0.5048	0.491	103.78	10	0.0791
Özkaynak [62]	103.20	0.5009	0.5048	103.70	10	0.1289
Aydin [63]	106.75	0.5043	0.4995	105.07	12	0.1289

Comparative results in Table 10 indicate that the proposed S-box provides a balanced cryptographic profile instead of performing well in only one parameter. It has an average nonlinearity of 112, which is the same as some of the strong S-boxes reported recently, such as Ali [40], Baowidan [42], Gulraiz [43], Abbasi [44], and Hazzazi [45], and is clearly higher than many other reported designs. More importantly, the proposed S-box also has BIC-NL = 112, DU = 4, and LAP = 0.0625, which are among the best values reported in the comparison. Mahboob [41] has a slightly higher average nonlinearity, but its DU and LAP values are less favorable than those of the proposed S-box. Likewise, some of the methods exhibit acceptable SAC or BIC-SAC values, but have higher DU or LAP values, which may make them more susceptible to differential or linear attacks. The average nonlinearity of the S-box after the refinement stage is 112, which is improved from 106 in the initial S-box; the DU of the S-box after the refinement stage is 4, which is reduced from 12 in the initial S-box; and the LAP of the S-box after the refinement stage is 0.0625, which is reduced from 0.1406 in the initial S-box. These enhancements demonstrate that the suggested group-action refinement is not just a rearrangement of the entries, but also significantly improves the cryptographic behavior of the S-box as a whole.

Table 10 also serves as an ablation analysis of the proposed two-stage framework. Stage 1 produces an algebraically structured and bijective S-box with average nonlinearity 106, BIC-NL 102.50, differential uniformity 16, and LAP 0.1406. After applying the Stage 2 group-action refinement, the average nonlinearity increases to 112, BIC-NL increases to 112, differential uniformity decreases from 12 to 4, and LAP decreases from 0.1406 to 0.0625. The BIC-SAC value also moves closer to the ideal value of 0.5. The SAC value changes only slightly, from 0.5017 to 0.5032, and remains close to the ideal value in both stages. These results show that Stage 1 supplies the algebraically generated bijective structure, while Stage 2 is responsible for the principal improvement in resistance to linear and differential cryptanalysis.

## 5. Proposed image encryption scheme

In all experiments reported in this paper, the number of encryption rounds is fixed at R=4. This setting is used for the Moon Surface, Airplane, and Clock images, including the statistical, differential, robustness, and execution-time experiments. The value R remains an explicit algorithm parameter, but no results for other round counts are claimed in this study

In order to demonstrate the practical utility of the proposed S-box, it is incorporated into a multi-round grayscale image-encryption scheme. In every round, the proposed S-box is used for substitution, followed by a key- and nonce-dependent position permutation and SHA-256-based keystream whitening. In the final round, a bidirectional nonlinear feedback-diffusion layer is applied to propagate changes throughout the ciphertext. A fresh 128-bit nonce is generated for each image and stored or transmitted with the ciphertext.

The encryption process is outlined in the following pseudocode.

**# Input:** Grayscale image `P`, Textual key `key_text`, Number of rounds `rounds`

**# Output:** Encrypted image `C` and the corresponding 128-bit nonce nonce

**# Initialization**

 i.         Load the proposed *8 × 8* S-box *`S`* (from Table 4).

 ii. Process `*key_text`* using SHA-256 to generate subkeys:

  *○ Kr*` (*16* bytes): For SHA-256-based keystream generation and diffusion.

  *○ Kp*` (*16* bytes): For key- and nonce-dependent position-permutation seeding.

 iii.  Flatten *`S`* into a 1D vector *Sflat, verify that it is bijective over the values 0–255, and use the proposed S-box in every encryption round.*

  iv.  Generate a fresh 128-bit nonce nonce for the input image using a cryptographically secure random generator. The nonce is not secret and is stored or transmitted with the encrypted image.

 v.    Generate the feedback digest as

feedback_digest = SHA256([Kr, nonce, 0]).

Derive three initialization bytes from this digest:

feedback = feedback_digest[1],

forward_IV = feedback_digest[2],

backward_IV = feedback_digest[3].

 The byte feedback is used for first-byte whitening in every round, whereas forward_IV and backward_IV initialize the final bidirectional nonlinear diffusion layer.

**# Encryption Process**

*Flatten the input image P into a 1D vector X.*

*For each round t = 1 to rounds, perform the following operations.*

**1. S-box Use:**

 ○ Use the proposed S-box vector Sflat in every round.

**2. Substitution:**

 ○ Substitute each pixel value *`v`* in *`X`* using the proposed *S-box:Y[i]=Sflat[X[i]+1].*

***3. Key- and Nonce-Dependent Position Permutation***

 *○ Compute a round-specific permutation digest using*

*permutation_digest = SHA256([Kp,nonce,t]).*

 *○ Use the first eight bytes of permutation_digest to obtain the pseudo-random generator seed.*

 *○ Convert these eight bytes to a 64-bit integer, reduce it modulo 2,147,483,647, and use the resulting nonzero value to seed the pseudo-random generator.*

 *○ Generate a pseudo-random permutation sequence gperm for the entire image-vector length:*

*gperm = RandPerm(length(Y), seedForRng).*

 *○ Apply the permutation:*

*U=Y[gperm].*

***4. Key- and Nonce-Dependent Keystream Generation***

 *○ Generate a pseudo-random keystream Z of the same length as U using the SHA-256-based PRNG with Kr, nonce, and the round index t:*

 *Z=SHA256_PRNG([Kr,nonce,t],length(U)).*

 *○ Combine the permuted vector with the round keystream:*

 *V[i]=U[i]⊕Z[i].*

 *○ Apply first-byte whitening in every round:*

 *V[1] = V[1] ⊕ feedback.*

***5. Round Update and Final Bidirectional Nonlinear Diffusion***

 *○ For rounds t < rounds, retain the substituted, permuted, and stream-whitened vector without applying a serial feedback chain:*

 *X = V.*

 *○ For the final round t = rounds, apply one bidirectional nonlinear diffusion layer.*

*Forward nonlinear diffusion*

 *○ Initialize the forward sequence as*

*F[1] = Sflat[(V[1] ⊕ forward_IV) + 1].*

 *○ For i = 2 to length(V), compute*

*F[i] = Sflat[(V[i] ⊕ F[i−1]) + 1].*

*Backward nonlinear diffusion*

 *○ Let L = length(V). Initialize the ciphertext sequence as*

*X[L] = Sflat[(F[L] ⊕ backward_IV) + 1].*

 *○ For i = L−1 down to 1, compute*

*X[i] = Sflat[(F[i] ⊕ X[i+1]) + 1].*

 ○ *This single forward-and-backward nonlinear diffusion layer propagates plaintext changes throughout the ciphertext while avoiding repeated serial diffusion in all four rounds.*

**# Finalize**

*After all rounds, reshape the final vector X back into a 2D matrix to form the encrypted image C.*

*Store or transmit the 128-bit nonce nonce together with C, because the same nonce is required to reconstruct the round permutations, keystreams, first-byte whitening value, and forward and backward diffusion initialization bytes during decryption.*

The decryption process is outlined in the following pseudocode.

*Input:*

 *Ciphertext image C*

 *Textual key key_text*

 *Number of rounds R = 4*

 *Stored 128-bit nonce*

*Output:*

 *Recovered grayscale image P*

*Initialisation:*

*1. Load the proposed S-box Sflat and construct its inverse Sinv:*

 *Sinv[Sflat[x] + 1] = x, for x = 0,...,255.*

*2. Apply the same key derivation used during encryption to obtain Kr and Kp.*

*3. Recompute:*

 *feedback_digest = SHA256([Kr, nonce, 0])*

 *feedback = feedback_digest[1]*

 *forward_IV = feedback_digest[2]*

 *backward_IV = feedback_digest[3]*

*4. Flatten C into the vector X and let L = length(X).*

*For t = R down to 1:*

  *A. Reverse the final bidirectional diffusion when t = R.*

  *Reverse the backward pass:*

   *F[L] = Sinv[X[L] + 1] XOR backward_IV*

   *For i = L-1 down to 1:*

  *F[i] = Sinv[X[i] + 1] XOR X[i+1]*

  *Reverse the forward pass:*

  *V[1] = Sinv[F[1] + 1] XOR forward_IV*

   *For i = 2 to L:*

  *V[i] = Sinv[F[i] + 1] XOR F[i-1]*

  *For t < R:*

   *V = X*

  *B. Remove first-byte whitening:*

   *V[1] = V[1] XOR feedback*

  *C. Regenerate the round keystream:*

   *Z = SHA256_PRNG([Kr, nonce, t], L)*

  *Remove stream whitening:*

   *U[i] = V[i] XOR Z[i], for i = 1,...,L*

  *D. Regenerate the round permutation:*

   *permutation_digest = SHA256([Kp, nonce, t])*

   *derive seedForRng exactly as in encryption*

   *gperm = RandPerm(L, seedForRng)*

  *Reverse U = Y[gperm]:*

   *For i = 1 to L:*

  *Y[gperm[i]] = U[i]*

  *E. Reverse S-box substitution:*

   *X[i] = Sinv[Y[i] + 1], for i = 1,...,L*

*Reshape X to the original image dimensions to obtain P.*

The decryption procedure is the exact inverse of the encryption procedure. The nonlinear diffusion is invertible because S is bijective and ${\text{S}}^{-\text{1}}$ is applied before removing each feedback value. The round permutation is inverted by assigning Y[gperm[i]]=U[i], and substitution is reversed using ${\text{S}}^{-\text{1}}$. For every unattacked test image, the recovered image is identical to the plaintext, giving mean squared error zero and PSNR = ∞. The public MATLAB implementation performs an exact-recovery check for each selected image and terminates with an error if decryption does not reproduce the original image exactly. The complete implementation is available at https://github.com/abdulrazaq-ship-it/Two-Stage-Algebraic-SBox-Image-Encryption.

### 5.1. Security considerations and key-space analysis

Let K denote the master key supplied to the scheme. If K is sampled uniformly from the set of 256-bit strings, the nominal brute-force key space is ${\text{2}}^{\text{2}\text{5}\text{6}}$. The subsequent SHA-256 processing derives the two 128-bit internal values $K_{r}$ and $K_{p}$. The public 128-bit nonce is not counted as part of the secret-key space. Its purpose is to ensure that the round permutations, keystreams, whitening byte, and diffusion initialisation values differ between encryptions performed under the same master key.

The statement ${\text{2}}^{\text{2}\text{5}\text{6}}$ applies only when the master key has 256 bits of genuine entropy. If key_text is a human-selected password, its effective security is limited by the entropy of that password; applying SHA-256 does not create additional entropy. A practical password-based implementation should therefore derive the master key using a standard salted password-based key-derivation function with an appropriate work factor, rather than applying a single unsalted SHA-256 operation.

A fresh nonce must be used for every image encrypted under the same master key. Reusing a key–nonce pair reproduces the same round-dependent permutations and keystream values and can expose relationships between messages. If 128-bit nonces are sampled uniformly, the approximate collision probability after q encryptions is $q(q-\text{1})/{\text{2}}^{\text{1}\text{2}\text{9}}$. Implementations should nevertheless store the nonce with the ciphertext and enforce uniqueness whenever feasible.

The proposed substitution, permutation, whitening, and bidirectional diffusion operations produce strong empirical avalanche and decorrelation behaviour in the reported experiments. The S-box has component nonlinearity 112 and differential uniformity 4, while the complete image scheme produces high NPCR and near-zero adjacent-pixel correlations. These results provide evidence against straightforward statistical and differential image analysis. They do not, however, constitute a formal proof of indistinguishability under chosen-plaintext or chosen-ciphertext attack.

The present construction also does not include an authentication tag. An attacker who modifies the ciphertext may cause uncontrolled changes in the recovered image without the receiver being able to distinguish malicious modification from transmission damage. For applications requiring both confidentiality and integrity, the ciphertext and nonce should be protected by a standard message-authentication mechanism or the construction should be placed inside a standard authenticated-encryption framework. Nonce management and authentication are fundamental requirements in authenticated-encryption designs.

**Fig 1:**
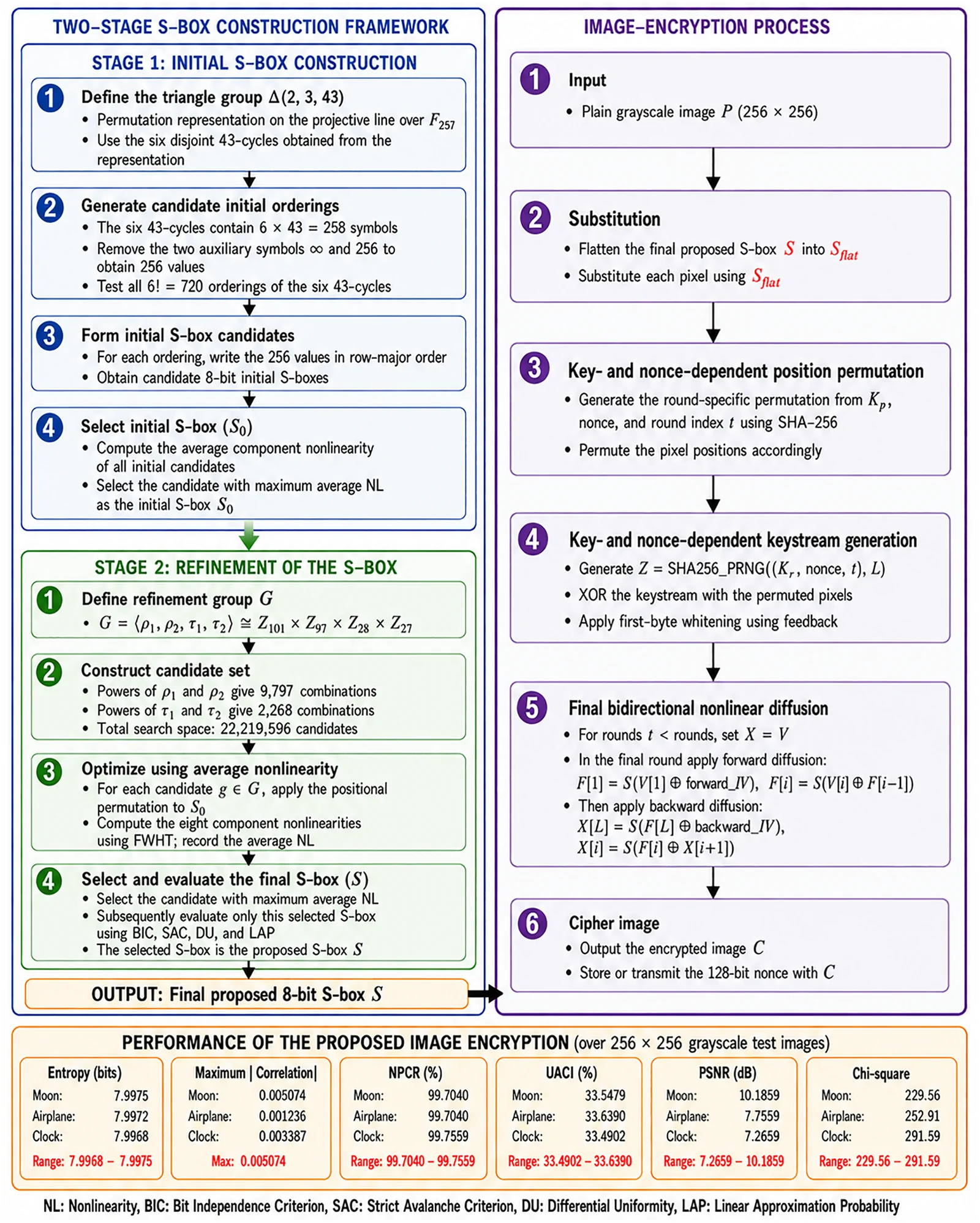
Flowchart of the proposed scheme.

Fig 1 presents two-stage S-box generation framework and image encryption workflow.

The experiments were conducted using three standard 256×256 grayscale benchmark images: were Moon Surface (5.1.09), Airplane (5.1.11), and Clock (5.1.12), obtained from the USC-SIPI Image Database. The images were processed at a resolution of 256×256 pixels. No human, animal, clinical, or privately collected data were used.

### 5.2. Statistical measures

This subsection discusses the effectiveness of the proposed encryption scheme using well-known statistical metrics. The statistical performance of the proposed scheme is compared with [64–68]. All three schemes were evaluated using the same three 256 × 256 images.

#### 5.2.1. Contrast.

Contrast measures local intensity variation in an image. Natural images usually exhibit moderate contrast due to structured regions. A secure encryption algorithm generates extremely high contrast, which means that pixel values have been reallocated in a highly chaotic fashion.

The Contrast is defined as:


$$\begin{array}{c} \text{Contrast}=\sum_{i=\text{0}}^{L-\text{1}}\sum_{j=\text{0}}^{L-\text{1}}\left|i-j{|}^{\text{2}}P(i,j\right) \end{array}$$

where p(i,j) is the normalized gray-level co-occurrence matrix (GLCM) and L is the number of gray levels.

#### 5.2.2. Homogeneity.

Homogeneity describes the resemblance of the neighboring pixels. Higher values represent smooth regions in the image. A substantial decrease in homogeneity after encryption is a proof that the cipher destroys local uniformity.

Homogeneity is defined as:


$$\begin{array}{c} \text{Homogeneity}=\sum_{i=\text{0}}^{L-\text{1}}\sum_{j=\text{0}}^{L-\text{1}}\frac{P(i,j)}{\text{1}+|i-j|}. \end{array}$$

#### 5.2.3. Energy.

Energy is used to measure uniformity of textures and patterns of repeated intensities. Natural images tend to have higher energy and encrypted images should have very low energy which demonstrates the removal of predictable structures.

Energy is defined as:


$$\begin{array}{c} \text{Energy}=\sum_{i=\text{0}}^{L-\text{1}}\sum_{j=\text{0}}^{L-\text{1}}P(i,j{)}^{\text{2}} \end{array}$$

#### 5.2.4. Entropy.

Entropy measures the uncertainty or randomness of distribution of pixel intensity. A value near 8 for an 8-bit image is an indication of excellent randomness and protection against statistical attacks.

Entropy is defined as:


$$\begin{array}{c} \text{Entropy}=-\sum_{i=\text{0}}^{L-\text{1}}p(k){\text{log}}_{\text{2}}p(k) \end{array}$$

where p(k) means the likelihood of occurrence of gray level k.

Table 11 summarizes the texture and statistical features of the original and encrypted images.

**Table 11:** Texture and statistical features of original and encrypted images.

Image	Scheme/Version	Contrast	Energy	Homogeneity	Entropy
Moon Surface	Original image	150.7682	0.00039467	0.219970	6.7093
	Proposed scheme	10953.0922	0.00002294	0.036388	7.9975
	[64]	10976.4160	0.00002294	0.036202	7.9972
	[65]	10963.8077	0.00002303	0.036205	7.9973
	[66]	10956.0947	0.00003164	0.035594	7.9659
	[67]	10874.4792	0.00002305	0.036577	7.9972
	[68]	10962.5344	0.00002299	0.035730	7.9973
Airplane	Original image	93.7550	0.00355214	0.465617	6.4523
	Proposed scheme	10938.5138	0.00002293	0.035972	7.9972
	[64]	10882.3750	0.00002298	0.036180	7.9971
	[65]	10851.3996	0.00002305	0.036637	7.9969
	[66]	10948.1284	0.00008979	0.034011	7.9525
	[67]	10966.2991	0.00002306	0.036102	7.9972
	[68]	10892.1771	0.00002304	0.036335	7.9972
Clock	Original image	285.6806	0.00502533	0.431925	6.7057
	Proposed scheme	10942.9537	0.00002304	0.036420	7.9968
	[64]	11023.3035	0.00002299	0.035729	7.9976
	[65]	10980.7945	0.00002296	0.036634	7.9965
	[66]	11074.6806	0.00014063	0.034479	7.9395
	[67]	10926.8984	0.00002298	0.036015	7.9971
	[68]	10891.4323	0.00002297	0.036196	7.9974

#### 5.2.5. Pixel correlation (Horizontal, Vertical, Diagonal).

Natural images tend to have high correlation between neighboring pixels. A strong image encryption algorithm should disrupt these spatial relationships, and therefore correlation values should approach zero in all directions. The correlation coefficientr between two adjacent pixels $x_{k}$ and $y_{k}$ is defined as:


$$\begin{array}{c} =\frac{\sum_{k=\text{1}}^{N}\left(x_{k}-\mu_{x}\right)\left(y_{k}-\mu_{y}\right)}{\sqrt{\sum_{k=\text{1}}^{N}{\left(x_{k}-\mu_{x}\right)}^{\text{2}}}\sqrt{\sum_{k=\text{1}}^{N}{\left(y_{k}-\mu_{y}\right)}^{\text{2}}}} \end{array}$$

where $x_{k}$ and $y_{k}$ are intensity values of adjacent pixel pairs, and also, $\mu_{x}$ and $\mu_{y}$ are their average values and N is the number of pixel pairs that are sampled.

In this study, the encrypted images showed minimal correlations:

For Moon Surface:

𝑟_𝐻_*=-0.002456*, 𝑟_𝑉_*=0.001613,* and 𝑟_𝐷_*=−0.005074*

For Airplane:

𝑟_𝐻_ *=0.000976, 𝑟*_*𝑉*_ *= −0.000982*, and 𝑟_𝐷_ *= −0.001236.*

For Clock:

𝑟_𝐻_ =*0.000720*, 𝑟_𝑉_=*0.003387*, and 𝑟_𝐷_ =*0.001243*.

Thus, all absolute correlation coefficients obtained for the proposed scheme are below 0.01, confirming effective decorrelation of adjacent pixels and strong diffusion.

#### 5.2.6. Chi-square Test.

This test checks how close the encrypted histogram is to a uniform distribution. Small Chi-square values show that each gray level occurs with almost equal probability, which prevents histogram-based attacks. It is computed as:


$$\begin{array}{c} \chi^{\text{2}}=\sum_{k=\text{0}}^{\text{2}\text{5}\text{5}}\frac{{\left(O_{k}-E_{k}\right)}^{\text{2}}}{E_{k}} \end{array}$$

where $O_{k}$ is the observed count of intensity k, and $E_{k}$ is the expected count under a uniform distribution.

#### 5.2.7. NPCR (Number of Pixels Change Rate).

NPCR evaluates how many pixel positions change when a single pixel in the plaintext is modified. A high NPCR (above 99 percent) indicates strong diffusion, making differential attacks ineffective. NPCR is defined as:


$$\begin{array}{c} NPCR=\frac{\text{1}}{MN}\sum_{i=\text{1}}^{M}\sum_{j=\text{1}}^{N}D(i,j)\times \text{1}\text{0}\text{0}\% \end{array}$$

where


$$\begin{array}{c} D(i,j)=\left\{ \begin{array}{c} @c\text{1},\text{ if }C_{\text{1}}(i,j)\neq C_{\text{2}}(i,j)\\ \text{0},\text{ otherwise} \end{array}\right. \end{array}$$

and $C_{\text{1}}$ and $C_{\text{2}}$ are two encrypted images produced from slightly different plaintexts.

#### 5.2.8. UACI (Unified Average Changing Intensity).

UACI measures the average intensity difference between two ciphertext images that originate from slightly different plaintexts. Values near 33 percent indicate that the encryption spreads changes widely and unpredictably. UACI is defined as:


$$\begin{array}{c} \text{UACI}=\frac{\text{1}}{MN}\sum_{i=\text{1}}^{M}\sum_{j=\text{1}}^{N}\frac{\left|C_{\text{1}}(i,j)-C_{\text{2}}(i,j)\right|}{\text{2}\text{5}\text{5}}\times \text{1}\text{0}\text{0}\% \end{array}$$

#### 5.2.9. PSNR (Peak Signal-to-Noise Ratio).

PSNR expresses the dissimilarity between the original and encrypted images. A low PSNR value confirms that the ciphertext bears no visual resemblance to the plaintext, which is desirable in encryption. PSNR is defined as:


$$\begin{array}{c} PSNR=\text{10 }{\text{log}}_{\text{1}\text{0}}\left(\frac{{\text{2}\text{5}\text{5}}^{\text{2}}}{MSE}\right) \end{array}$$

with


$$\begin{array}{c} MSE=\frac{\text{1}}{MN}\sum_{i=\text{1}}^{M}\sum_{j=\text{1}}^{N}{\left(I(i,j)-C(i,j)\right)}^{\text{2}} \end{array}$$

where I(i,j) is the original pixel value, and C(i,j) is the encrypted pixel value.

Table 12 reports the correlation, chi-square, NPCR, UACI, and PSNR results of the encrypted images.

**Table 12:** Correlation, Chi-square, NPCR, UACI, and PSNR of encrypted images.

*Image*	*Scheme*	*Pixel Corr. H*	*Pixel Corr. V*	*Pixel Corr. D*	*Chi-Square (Enc.)*	*NPCR (%)*	*UACI (%)*	*PSNR (dB)*
***Moon Surface***	*Proposed scheme*	*−0.002456*	*0.001613*	*−0.005074*	*229.56*	*99.7040*	*33.5479*	*10.1859*
	*[64]*	*−0.005594*	*−0.001925*	*0.002688*	*255.38*	*99.5193*	*33.5026*	*10.1725*
	*[65]*	*−0.007407*	*0.007289*	*0.001294*	*242.06*	*99.6338*	*33.5603*	*10.2208*
	*[66]*	*−0.003874*	*−0.002685*	*−0.004382*	*2936.23*	*0.0015*	*0.0004*	*10.1780*
	*[67]*	*0.004964*	*−0.006315*	*0.008002*	*257.16*	*93.3960*	*30.2035*	*10.1865*
	*[68]*	*−0.004740*	*−0.006578*	*−0.001900*	*247.19*	*99.6078*	*33.4712*	*10.1816*
***Airplane***	*Proposed scheme*	*0.000976*	*−0.000982*	*−0.001236*	*252.91*	*99.7040*	*33.6390*	*7.7559*
	*[64]*	*−0.000653*	*−0.008614*	*0.004777*	*258.28*	*99.5987*	*33.3646*	*7.7644*
	*[65]*	*0.006634*	*0.040610*	*−0.004447*	*281.21*	*99.5865*	*33.6587*	*7.7175*
	*[66]*	*−0.014194*	*−0.010269*	*−0.010604*	*4397.86*	*0.0015*	*0.0004*	*7.7664*
	*[67]*	*−0.004673*	*0.004639*	*0.004640*	*254.70*	*93.3960*	*30.1241*	*7.7322*
	*[68]*	*0.002597*	*−0.001938*	*0.000507*	*253.64*	*99.6170*	*33.5290*	*7.7340*
***Clock***	*Proposed scheme*	*0.000720*	*0.003387*	*0.001243*	*291.59*	*99.7559*	*33.4902*	*7.2659*
	*[64]*	*−0.003375*	*0.003985*	*−0.001717*	*217.09*	*99.5987*	*33.4467*	*7.2833*
	*[65]*	*−0.001046*	*0.022123*	*−0.004580*	*319.35*	*99.6048*	*33.2360*	*7.2556*
	*[66]*	*−0.022769*	*−0.027574*	*−0.001505*	*5927.05*	*0.0015*	*0.0008*	*7.2284*
	*[67]*	*−0.000272*	*−0.003846*	*−0.001561*	*265.95*	*93.3960*	*30.2465*	*7.2658*
	*[68]*	*0.004468*	*−0.000898*	*−0.004190*	*233.63*	*99.6216*	*33.4676*	*7.2822*

### 5.3. Robustness against cropping and noise attacks

In order to further investigate the practical security of the proposed image encryption scheme, the scheme was tested under cropping and noise attacks. In these experiments, the encrypted images were first subjected to different attack levels and were then decrypted using the correct secret key. The quality of the recovered images was measured using peak signal-to-noise ratio (PSNR), structural similarity index measure (SSIM), and mean absolute error (MAE). The original encrypted images without attack were decrypted perfectly, with PSNR =∞, which confirms the correctness of the encryption and decryption process.

Table 13 reports the results under cropping attacks of 6.25%, 12.50%, and 25.00%. As expected, the reconstruction quality decreases when the cropped area becomes larger. However, the decrypted images can still be recovered with reasonable quality under mild cropping, particularly at 6.25%. This means that the proposed scheme shows a certain degree of tolerance against partial data loss, although heavier cropping naturally results in greater degradation in the recovered image quality.

**Table 13:** Robustness analysis of the proposed scheme under cropping attacks.

Image	Cropping Level	PSNR (dB)	SSIM	MAE
Moon Surface	6.25%	21.9813	0.4865	4.3595
Moon Surface	12.50%	18.9755	0.2926	8.7515
Moon Surface	25.00%	16.1000	0.1575	17.0929
Airplane	6.25%	19.7295	0.3206	5.4527
Airplane	12.50%	16.6949	0.1753	10.9484
Airplane	25.00%	13.6843	0.0970	21.7296
Clock	6.25%	19.2017	0.3903	5.7969
Clock	12.50%	16.2216	0.2474	11.5713
Clock	25.00%	13.2354	0.1538	22.9026

Table 14 shows the results for Gaussian and salt-and-pepper noise attacks. Gaussian noise results in low PSNR and SSIM values as it changes a significant number of the ciphertext pixels, and the errors are compounded during the decryption process. By contrast, salt-and-pepper noise at density 0.0100 allows significantly better recovery, with PSNR values between 22.3883 and 25.5915 dB, SSIM values between 0.5304 and 0.7047, and MAE values between 1.9413 and 2.7936. When the density increases to 0.0500, the PSNR decreases to 15.6581–18.5998 dB and the MAE increases to 9.6674–13.0807. These results show that the quality of the reconstruction is consistently reduced as the noise level increases.

**Table 14:** Robustness analysis of the proposed scheme under gaussian and salt-and-pepper noise attacks.

Image	Attack type	Attack level	PSNR (dB)	SSIM	MAE
Moon Surface	Gaussian noise	Variance 0.0010	10.1690	0.0448	67.2092
Moon Surface	Gaussian noise	Variance 0.0050	10.1851	0.0443	67.0008
Moon Surface	Salt-and-pepper noise	Density 0.0100	25.5915	0.7047	1.9413
Moon Surface	Salt-and-pepper noise	Density 0.0500	18.5998	0.2694	9.6674
Airplane	Gaussian noise	Variance 0.0010	7.7479	0.0280	85.2868
Airplane	Gaussian noise	Variance 0.0050	7.7616	0.0279	85.2683
Airplane	Salt-and-pepper noise	Density 0.0100	22.8569	0.5304	2.6446
Airplane	Salt-and-pepper noise	Density 0.0500	16.1803	0.1580	12.2150
Clock	Gaussian noise	Variance 0.0010	7.3157	0.0380	89.6922
Clock	Gaussian noise	Variance 0.0050	7.2816	0.0380	90.1344
Clock	Salt-and-pepper noise	Density 0.0100	22.3883	0.5860	2.7936
Clock	Salt-and-pepper noise	Density 0.0500	15.6581	0.2293	13.0807

These findings complement the statistical and differential analyses already reported in Section 5. The proposed encryption scheme is lossless without any attack and is stable under cropping and noise attacks. The results also show a clear trend: the smaller the attack on the image, the lower the distortion in the recovered image; conversely, the stronger the attack, either by cropping or added noise, the greater the distortion in the recovered image, which is expected. Therefore, the further analysis shows that the proposed scheme has useful robustness against common image degradation attacks and has good statistical security.

### 5.4. Discussion of the statistical security and robustness of the proposed image encryption scheme

The numerical results in Table 11 show that the evaluated schemes substantially disrupt the texture characteristics of the plaintext images. For the proposed scheme, the encrypted-image contrast values are 10953.0922, 10938.5138, and 10942.9537 for Moon Surface, Airplane, and Clock, respectively. The corresponding energy values remain very small, ranging from 0.00002293 to 0.00002304, while the homogeneity values range from 0.035972 to 0.036420. The ciphertext entropy values are 7.9975, 7.9972, and 7.9968, respectively, and are therefore very close to the ideal value of 8 for an 8-bit grayscale image. These results show that there is significant destruction of the plaintext texture and a high level of randomness in the ciphertext. The five reference schemes [64]–[68] also have different levels of statistical randomization, which makes it possible to evaluate the proposed scheme against a wider range of recent schemes.

Table 12 also shows the statistical and differential performance of the proposed scheme. All the horizontal, vertical, and diagonal adjacent-pixel correlation coefficients are less than 0.006, and the maximum absolute value is 0.005074. This validates the good decorrelation of the adjacent pixels of the encrypted images. The proposed scheme produces NPCR values of 99.7040%, 99.7040%, and 99.7559% for Moon Surface, Airplane, and Clock, respectively, while the corresponding UACI values are 33.5479%, 33.6390%, and 33.4902%. These UACI values are near the expected value of around 33.46% for 8-bit image encryption. The PSNR values of the ciphertext images vary from 7.2659 dB to 10.1859 dB, which shows that there is a significant difference between the original and the encrypted images. The chi-square values are 229.56, 252.91, and 291.59 for the three test images. In general, the proposed scheme has near-zero adjacent-pixel correlation, high NPCR values, UACI values near the expected benchmark, and high ciphertext entropy for all three images.

As seen in Table 13, the quality of the recovered images is degraded gradually as the cropped area is increased. For Moon Surface, the PSNR decreases from 21.9813 dB at 6.25% cropping to 18.9755 dB at 12.50% and 16.1000 dB at 25.00%, while the MAE increases from 4.3595 to 17.0929. The same is true for Airplane and Clock. Across the three images, the PSNR values range from 19.2017 to 21.9813 dB at 6.25% cropping and from 13.2354 to 16.1000 dB at 25.00% cropping. At the same time, the SSIM values decrease and the MAE values increase as the cropped region becomes larger. These results demonstrate graceful degradation: useful visual information remains recoverable under mild cropping, whereas more substantial data loss results in greater reconstruction distortion.

The noise-attack results in Table 14 show a consistent relationship between attack severity and reconstruction quality. Under Gaussian noise, the PSNR values range from 7.2816 to 10.1851 dB, the SSIM values range from 0.0279 to 0.0448, and the MAE values range from 67.0008 to 90.1344. These relatively weak reconstruction results occur because Gaussian noise affects a large proportion of ciphertext pixels and the resulting disturbances propagate during decryption. Salt-and-pepper noise at density 0.0100 permits substantially better recovery, producing PSNR values of 25.5915 dB for Moon Surface, 22.8569 dB for Airplane, and 22.3883 dB for Clock. The corresponding SSIM values range from 0.5304 to 0.7047, while the MAE values remain between 1.9413 and 2.7936. When the density increases to 0.0500, the PSNR decreases to 18.5998, 16.1803, and 15.6581 dB, respectively, and the MAE increases to between 9.6674 and 13.0807. These results confirm that reconstruction quality decreases as the noise intensity increases. The proposed image encryption scheme provides exact recovery when no attack is applied and demonstrates measurable tolerance to cropping and noise disturbances, with reconstruction quality decreasing progressively as attack severity increases.

### 5.5. Computational complexity and practical efficiency

The computational cost of the framework can be divided into the S-box construction and the image-encryption process. During Stage 1, all 6!=720 arrangements of the six 43-cycles are evaluated. For an m-bit S-box, independently computing the nonlinearities of its m coordinate functions using the fast Walsh–Hadamard transform requires $O(m^{\text{2}}.{\text{2}}^{m})$ operations per candidate. The direct worst-case complexity of the complete Stage 2 search is therefore $O\left(|G|.m^{\text{2}}.{\text{2}}^{m}\right)$, where m=8 and ∣G∣=22,219,596.

To reduce repeated computation, the implementation exploits the direct-product structure of the refinement group. The powers of a and b produce $N_{AB}=\text{1}\text{0}\text{1}\times \text{9}\text{7}=\text{9},\text{7}\text{9}\text{7}$ combinations, while the powers of c,d, and e produce $N_{CDE}=\text{2}\text{7}\times \text{2}\text{8}\times \text{3}=\text{2},\text{2}\text{6}\text{8}$ combinations. Their product is $N_{AB}.N_{CDE}=\text{2}\text{2},\text{2}\text{1}\text{9},\text{5}\text{9}\text{6},$ so the complete group is still searched exhaustively. Walsh contributions for the AB and CDE parts are precomputed and reused when the two parts are combined. The precomputation requires $O\left(\left(N_{AB}+N_{CDE}\right)m{.\text{2}}^{m}\right)$ operations, while the subsequent exhaustive combination stage has worst-case complexity $O(N_{AB}.N_{CDE}.m{.\text{2}}^{m})=O\left(|G|.m.{\text{2}}^{m}\right).$

Pruning reduces the average number of operations but does not omit any group element from the search.

The program was executed in MATLAB on an HP Pavilion Aero 13 laptop equipped with an AMD Ryzen 78840U processor operating at approximately 3.30 GHz, with 8 physical cores, 16 logical processors, and 16 GB RAM. Walsh-contribution precomputation required 0.362 s, and the exhaustive search over all 22,219,596 candidates required 25.069 s. The total reported core execution time was 25.431 s, giving a search rate of approximately 0.886 million candidates per second. The search returned the element $a^{\text{7}\text{5}}b^{\text{9}\text{5}}c^{\text{1}\text{1}}d^{\text{2}\text{3}}e^{\text{2}}$, whose eight component nonlinearities are all equal to 112.

The exhaustive search is a one-time operation. Once the final S-box has been selected, it is stored as a 256-entry lookup table and is used directly during encryption and decryption. Consequently, the refinement cost does not affect the per-image execution time. Table 15 summarizes the computational performance of the Stage 2 refinement.

**Table 15:** Computational performance of the Stage 2 refinement.

Measure	Value
AB combinations	9,797
CDE combinations	2,268
Complete search space	22,219,596
Walsh precomputation time	0.362 s
Exhaustive search time	25.069 s
Total reported core time	25.431 s
Search rate	0.886 million candidates/s
Processor	AMD Ryzen 7 8840U
Processor configuration	8 cores, 16 logical processors
Installed RAM	16 GB
Selected group element	$a^{\text{7}\text{5}}b^{\text{9}\text{5}}c^{\text{1}\text{1}}d^{\text{2}\text{3}}e^{\text{2}}$
Average/minimum NL	112/112

Table 16 reports the execution performance obtained over 30 runs for each 256 × 256 grayscale image. The mean median encryption and decryption times are 0.002656 s and 0.002487 s, respectively. The corresponding mean throughputs are 25.131 MP/s for encryption and 26.894 MP/s for decryption. These results show that the computationally expensive group-action search is confined to the offline S-box construction phase. Once the final S-box has been selected and stored as a 256-entry lookup table, the online cipher performs substitution, key- and nonce-dependent permutation, SHA-256-based keystream generation, XOR operations, and the final bidirectional nonlinear diffusion. Consequently, a 256 × 256 grayscale image can be encrypted or decrypted within a few milliseconds on the tested platform.

**Table 16:** Encryption and decryption performance for 256×256 grayscale images.

Image	Encryption time, median (s)	Decryption time, median (s)	Encryption throughput (MP/s)	Decryption throughput (MP/s)
Moon Surface	0.002501	0.002359	26.200	27.781
Airplane	0.002299	0.002118	28.505	30.946
Clock	0.003168	0.002985	20.687	21.956
Mean	0.002656	0.002487	25.131	26.894

The proposed framework is also suitable for parallel implementation. During Stage 2, the candidate S-box associated with one group element can be evaluated independently of the candidates associated with other group elements. The group elements can therefore be divided among multiple CPU cores, computing nodes, or GPU batches. Walsh-spectrum calculations and the construction of DDT and LAT tables can also be vectorized. In the online cipher, S-box lookup, keystream generation, XOR operations, and index rearrangement can be performed in parallel. The Stage 2 candidate evaluations and several independent operations can be parallelized. However, the nonlinear feedback diffusion contains sequential data dependencies and is therefore less directly amenable to parallel execution. The present study reports a CPU-based MATLAB implementation; a dedicated GPU, multicore, or FPGA implementation and the resulting speedup will be investigated in future work.

## 5. Conclusion

A new, group-theoretic method of constructing cryptographically strong S-boxes has been introduced in this paper. The approach uses a parameterized permutation representation of the triangle group Δ(2,3,43) over a finite field to generate a structurally sound initial S-box. The most important innovation is the subsequent refinement process, the action of permutation group $C_{\text{7406532}}\times C_{\text{3}}$ is applied on initial S-box. The refinement raises the average nonlinearity to 112. Extensive cryptographic tests verify that it is resistant to linear, differential, and statistical attacks.

Moreover, the proposed S-box being incorporated into a suggested image encryption scheme highlights its feasibility. Ciphertexts generated by the proposed encryption algorithm, which combines fixed S-box substitution, key- and nonce-dependent position permutation, SHA-256-based keystream whitening, and final bidirectional nonlinear feedback diffusion, exhibit high randomness, near-zero adjacent-pixel correlation, and strong sensitivity to plaintext changes. Further cropping and noise experiments are also conducted to show that the scheme is stable under common ciphertext degradation attacks, and reconstruction quality decreases gradually with the increasing attack level. Comparison with the five reference schemes demonstrates that the proposed method is competitive across the evaluated texture and statistical measures. In particular, it achieves consistently high NPCR values across all three test mages while maintaining near-zero adjacent-pixel correlations and UACI values close to the expected benchmark.

The present study has several limitations. First, the algebraic investigation is limited to one triangle group, one finite field, and one selected admissible parameter tuple. Other valid parameter choices may generate initial S-boxes with different cryptographic properties. Second, the image experiments are limited to three 256×256 grayscale images, and the current implementation has not been evaluated on color images, high-resolution images, video data, embedded hardware, or side-channel attack models.

Future work will examine alternative admissible parameter sets and triangle groups, lightweight and larger S-boxes, authenticated image encryption, color and video data, GPU and FPGA implementations, and implementation-level resistance to timing, power-analysis, and fault attacks.

The construction principle is extensible to other S-box dimensions, although such an extension is not obtained by simply resizing the present S-box. A lightweight 4-bit construction may, for example, be investigated over $F_{\text{1}\text{7}}$, while $F_{\text{65537}}$ provides a possible starting point for a 16-bit construction under the same projective-line principle. In each case, a suitable triangle-group representation, an admissible parameter set, and a new refinement group must be constructed and verified. Lightweight 4-bit or compact 8-bit versions may be relevant to IoT devices, but their gate area, memory use, latency, energy consumption, and side-channel behavior would have to be evaluated. For larger S-boxes, exhaustive refinement becomes increasingly expensive, and parallel, heuristic, or multi-stage screening methods would be required. Thus, the present framework provides a general construction strategy, while practical scalability remains dependent on the target S-box size and implementation platform.

## Supporting information

S1 AppendixPositional action of the Stage 2 refinement permutation on the initial S-box.This appendix gives the disjoint cycle decompositions of the generators a,b,c,d and e of the refinement group $C_{\text{7}\text{4}\text{0}\text{6}\text{5}\text{3}\text{2}}\times C_{\text{3}},$ states the combined cycle decomposition of the selected element $a^{\text{7}\text{5}}b^{\text{9}\text{5}}c^{\text{1}\text{1}}d^{\text{2}\text{3}}e^{\text{2}}$, and illustrates how this element rearranges the 256 positions of the initial S-box to produce the proposed S-box reported in Table 4.(DOCX)

## References

[1] Molinaro CA, Ruaro RL. Privacy protection with regard to (tele-)communications surveillance and data retention. In: Ius Gentium: Comparative Perspectives on Law and Justice. Springer International Publishing; 2022. pp. 113–32. doi: 10.1007/978-3-030-90331-2_6

[2] Mollin RA. An introduction to cryptography. Boca Raton, FL, USA: CRC Press; 2000.

[3] Hamad S, Rashid OF, Al-Dabbas HM. Text cryptography and steganography based on RNA sequences generation, true random number generators, and codebook method. PLoS One. 2025;20(12):e0338700. doi: 10.1371/journal.pone.0338700 41364697 PMC12688134

[4] Omar O, Tuama SA, Mohammed IJ, Subhi MA. AI-driven cryptographic and steganographic integration for enhanced text security using OpenAI API. FPA. 2025;19(1):108–16. doi: 10.54216/fpa.190110

[5] Wang X, Wang X, Zhao J, Zhang Z. Chaotic encryption algorithm based on alternant of stream cipher and block cipher. Nonlinear Dyn. 2010;63(4):587–97. doi: 10.1007/s11071-010-9821-4

[6] Zhang T, Chen CLP, Chen L, Xu X, Hu B. Design of highly nonlinear substitution boxes based on I-Ching operators. IEEE Trans Cybern. 2018;48(12):3349–58. doi: 10.1109/TCYB.2018.2846186 30040668

[7] Biham E, Shamir A. Differential cryptanalysis of DES-like cryptosystems. J Cryptology. 1991;4(1):3–72. doi: 10.1007/bf00630563

[8] Anees A, Ahmed Z. A technique for designing substitution box based on van der pol oscillator. Wireless Pers Commun. 2015;82(3):1497–503. doi: 10.1007/s11277-015-2295-4

[9] Ullah A, Jamal SS, Shah T. A novel construction of substitution box using a combination of chaotic maps with improved chaotic range. Nonlinear Dyn. 2017;88(4):2757–69. doi: 10.1007/s11071-017-3409-1

[10] Özkaynak F. Construction of robust substitution boxes based on chaotic systems. Neural Comput & Applic. 2017;31(8):3317–26. doi: 10.1007/s00521-017-3287-y

[11] Shafique A. A new algorithm for the construction of substitution box by using chaotic map. Eur Phys J Plus. 2020;135(2). doi: 10.1140/epjp/s13360-020-00187-0

[12] Ali A, Khan MA, Ayyasamy RK, Wasif M. A novel systematic byte substitution method to design strong bijective substitution box (S-box) using piece-wise-linear chaotic map. PeerJ Comput Sci. 2022;8:e940. doi: 10.7717/peerj-cs.940 35634109 PMC9138039

[13] Liu H, Liu J, Ma C. Constructing dynamic strong S-Box using 3D chaotic map and application to image encryption. Multimed Tools Appl. 2023;82(16):23899–914.

[14] Ullah S, Liu X, Waheed A, Zhang S. An efficient construction of S-box based on the fractional-order Rabinovich–Fabrikant chaotic system. Integration. 2024;94:102099. doi: 10.1016/j.vlsi.2023.102099

[15] Tolpa SH, Abdelhamed MA, Said E-SSA, Afi MYI. A novel chaos-based approach for constructing lightweight S-Boxes. Sci Rep. 2025;15(1):34112. doi: 10.1038/s41598-025-20019-4 41028281 PMC12485111

[16] Razaq A, Alolaiyan H, Ahmad M, Yousaf MA, Shuaib U, Aslam W, et al. A Novel method for generation of strong substitution-boxes based on coset graphs and symmetric groups. IEEE Access. 2020;8:75473–90. doi: 10.1109/access.2020.2989676

[17] Razaq A, Akhter S, Yousaf A, Shuaib U, Ahmad M. A group theoretic construction of highly nonlinear substitution box and its applications in image encryption. Multimed Tools Appl. 2021;81(3):4163–84. doi: 10.1007/s11042-021-11635-z

[18] Arshad B, Siddiqui N, Hussain Z, Ehatisham-ul-Haq M. A novel scheme for designing secure substitution boxes (S-boxes) based on Mobius group and finite field. Wireless Pers Commun. 2022;124(4):3527–48.

[19] Arshad S. Construction of 4× 4 substitution box using elliptic curves and algebraic group structures. Wireless Pers Commun. 2023;131(3):1913–27.

[20] Razaq A, Alhamzi G, Abbas S, Ahmad M, Razzaque A. Secure communication through reliable S-box design: A proposed approach using coset graphs and matrix operations. Heliyon. 2023;9(5):e15902. doi: 10.1016/j.heliyon.2023.e15902 37215757 PMC10192417

[21] Tita F, Setiawan A, Susanto B. Construction of substitution box (s-box) based on irreducible polynomials on gf (2^ 8). Bareken J Ilmu Mat Terapan. 2024;18(1):0517–28.

[22] Ahmad M, Bhatia D, Hassan Y. A novel ant colony optimization based scheme for substitution box design. Procedia Comput Sci. 2015;57:572–80. doi: 10.1016/j.procs.2015.07.394

[23] Picek S, Cupic M, Rotim L. A new cost function for evolution of S-boxes. Evol Comput. 2016;24(4):695–718. doi: 10.1162/EVCO_a_00191 27482748

[24] Zahid AH, Iliyasu AM, Ahmad M, Shaban MMU, Arshad MJ, Alhadawi HS, et al. A novel construction of dynamic S-Box with high nonlinearity using heuristic evolution. IEEE Access. 2021;9:67797–812. doi: 10.1109/access.2021.3077194

[25] Shadab M, Jawed MS, Sajid M. Substitution box construction using transfer‐function assisted metaheuristic and booster algorithm: a hybrid approach. Secur Priv. 2025;8(1):e462.

[26] Jia C, Cui T, Ling Q, He Y, Hu K, Sun Y, et al. How small can S-boxes be?. ToSC. 2025;2025(1):592–622. doi: 10.46586/tosc.v2025.i1.592-622

[27] Artuğer F, Özkaynak F. An effective method to improve nonlinearity value of substitution boxes based on random selection. Inf Sci. 2021;576:577–88. doi: 10.1016/j.ins.2021.07.036

[28] Jawed MS, Sajid M. COBLAH: A chaotic OBL initialized hybrid algebraic-heuristic algorithm for optimal S-box construction. Comput Stand Interfaces. 2025;91:103890. doi: 10.1016/j.csi.2024.103890

[29] Rashid OF, Subhi MA, Hussein MK, Mahdi MN. Enhancing internet data security: a fusion of cryptography, steganography, and machine learning techniques. Baghdad SciJ. 2024. doi: 10.21123/bsj.2024.10371

[30] Artuğer F. Strong s-box construction approach based on Josephus problem. Soft Comput. 2024;28(17–18):10201–13. doi: 10.1007/s00500-024-09751-7

[31] Rashid OF, Abdulsahib SA, Al-Shahwani H. Text encryption through bio-inspired DNA and RNA sequencing. PLoS One. 2026;21(4):e0345090. doi: 10.1371/journal.pone.0345090 41950265 PMC13061171

[32] Alali AS, Jamil MK, Ali R, Alotaibi R, Albalawi W. Degree, closeness and eigenvector for the construction of cryptographically secure S-boxes. Ain Shams Engineering Journal. 2025;16(10):103559. doi: 10.1016/j.asej.2025.103559

[33] Song Q, Li L, Yan L. Construction of lightweight s-boxes with low boomerang uniformity. IEEE Trans Circuits Syst I. 2026;73(2):1183–95. doi: 10.1109/tcsi.2025.3615643

[34] Rashid OF, Tuama SA, Al-Shahwani H. A multi-image codebook approach for secure text transmission. PLoS One. 2025;20(12):e0338836. doi: 10.1371/journal.pone.0338836 41385575 PMC12700361

[35] Qu S, Shi Q, An X. A novel heterogeneous coupled neuron model and its application in medical image encryption. Nonlinear Dyn. 2026;114(5). doi: 10.1007/s11071-026-12263-8

[36] Yu F, Wang X, Guo R, Ying Z, Cai S, Jin J. Dynamical analysis, hardware implementation, and image encryption application of new 4D discrete hyperchaotic maps based on parallel and cascade memristors. Integration. 2025;104:102475. doi: 10.1016/j.vlsi.2025.102475

[37] Jin J, Wu M, Ouyang A, Li K, Chen C. A novel dynamic hill cipher and its applications on medical IoT. IEEE Internet Things J. 2025;12(10):14297–308. doi: 10.1109/jiot.2025.3525623

[38] Du X, Zhu J, Zhou J-Z, Pun C-M, Lin Z, Wu C, et al. DP-TRAE: a dual-phase merging transferable reversible adversarial example for image privacy protection. IEEE Trans Dependable and Secure Comput. 2025;22(6):7849–61. doi: 10.1109/tdsc.2025.3601175

[39] Mushtaq Q. Parametrization of all homomorphisms from PGL(2,Z) into PGL(2,q). Commun Algebra. 1992;20(4):1023–40.

[40] Ali J, Jamil MK, Alali AS, Ali R, Gulraiz. A medical image encryption scheme based on Mobius transformation and Galois field. Heliyon. 2023;10(1):e23652. doi: 10.1016/j.heliyon.2023.e23652 38192806 PMC10772117

[41] Mahboob A. A mathematical approach for generating a highly non-linear substitution box using quadratic fractional transformation. CMC. 2023;77(2):2565–78. doi: 10.32604/cmc.2023.040371

[42] Baowidan SA, Alamer A, Hassan M, Yousaf A. Group-action-based S-box generation technique for enhanced block cipher security and robust image encryption scheme. Symmetry. 2024;16(8):954. doi: 10.3390/sym16080954

[43] Gulraiz RA, Ali J. Enhancing visual privacy: a novel RGB image encryption method employing Galois field. Open J Discrete Appl Math. 2025;8(3):93–108. doi: 10.30538/psrp-odam2025.0124

[44] Abbasi AZ, Rafiq A, Jaghdam IH, Maatki C, Hassen W, Alshammari BM. Generalized triangle group based S-box construction for secure image encryption. Sci Rep. 2025;15(1):38608. doi: 10.1038/s41598-025-22558-2 41188397 PMC12586604

[45] Mazyad Hazzazi M, Gulraiz Ali R, Kamran Jamil M, Abdullah Nooh S, Alblehai F. Cryptanalysis of hyperchaotic S-box generation and image encryption. MATH. 2024;9(12):36116–39. doi: 10.3934/math.20241714

[46] Ahmed HA, Zolkipli MF, Ahmad M. A novel efficient substitution-box design based on firefly algorithm and discrete chaotic map. Neural Comput & Applic. 2018;31(11):7201–10. doi: 10.1007/s00521-018-3557-3

[47] Hayat U, Azam NA, Gallegos-Ruiz HR, Naz S, Batool L. A Truly dynamic substitution box generator for block ciphers based on elliptic curves over finite rings. Arab J Sci Eng. 2021;46(9):8887–99. doi: 10.1007/s13369-021-05666-9

[48] Ibrahim S, Abbas AM. Efficient key-dependent dynamic S-boxes based on permutated elliptic curves. Inf Sci. 2021;558:246–64. doi: 10.1016/j.ins.2021.01.014

[49] Alshammari BM, Guesmi R, Guesmi T, Alsaif H, Alzamil A. Implementing a symmetric lightweight cryptosystem in highly constrained IoT devices by using a chaotic S-box. Symmetry. 2021;13(1):129. doi: 10.3390/sym13010129

[50] Alhadawi HS, Majid MA, Lambić D, Ahmad M. A novel method of S-box design based on discrete chaotic maps and cuckoo search algorithm. Multimed Tools Appl. 2020;80(5):7333–50. doi: 10.1007/s11042-020-10048-8

[51] Long M, Wang L. S-box design based on discrete chaotic map and improved artificial bee colony algorithm. IEEE Access. 2021;9:86144–54. doi: 10.1109/access.2021.3069965

[52] Soto R, Crawford B, Molina FG, Olivares R. Human behaviour based optimization supported with self-organizing maps for solving the S-box design problem. IEEE Access. 2021;9:84605–18. doi: 10.1109/access.2021.3087139

[53] Yan W, Ding Q. A novel S-box dynamic design based on nonlinear-transform of 1D chaotic maps. Electronics. 2021;10(11):1313. doi: 10.3390/electronics10111313

[54] Zhou P, Du J, Zhou K, Wei S. 2D mixed pseudo-random coupling PS map lattice and its application in S-box generation. Nonlinear Dyn. 2021;103(1):1151–66. doi: 10.1007/s11071-020-06098-0

[55] Jamal SS, Hazzazi MM, Khan MF, Bassfar Z, Aljaedi A, ul Islam Z. Region of interest-based medical image encryption technique based on chaotic S-boxes. Expert Syst Appl. 2024;238:122030. doi: 10.1016/j.eswa.2023.122030

[56] Wang M, Liu H, Zhao M. Construction of a non-degeneracy 3D chaotic map and application to image encryption with keyed S-box. Multimed Tools Appl. 2023;82(22):34541–63. doi: 10.1007/s11042-023-14988-9

[57] Ejaz A, Murtaza G, Haider T, Azam NA, Hayat U. An Efficient S‐box generation with mutation optimization for secure image encryption. Security and Privacy. 2025;8(4). doi: 10.1002/spy2.70068

[58] Zamli KZ, Din F, Alhadawi HS. Exploring a Q-learning-based chaotic naked mole rat algorithm for S-box construction and optimization. Neural Comput & Applic. 2023;35(14):10449–71. doi: 10.1007/s00521-023-08243-3

[59] Murtaza G, Hayat U. Efficient image encryption algorithm based on ECC and dynamic S-box. J Inf Secur Appl. 2025;90:104004. doi: 10.1016/j.jisa.2025.104004

[60] Farah T, Rhouma R, Belghith S. A novel method for designing S-box based on chaotic map and teaching–learning-based optimization. Nonlinear Dyn. 2017;88(2):1059–74.

[61] Alhadawi HS, Lambić D, Zolkipli MF, Ahmad M. Globalized firefly algorithm and chaos for designing substitution box. J Inf Secur Appl. 2020;55:102671. doi: 10.1016/j.jisa.2020.102671

[62] Özkaynak F, Özer AB. A method for designing strong S-boxes based on chaotic lorenz system. Physics Letters A. 2010;374(36):3733–8. doi: 10.1016/j.physleta.2010.07.019

[63] Aydin Y, Garipcan AM, Özkaynak F. Correction to: a novel Secure S-box design methodology based on fpga and sha-256 hash algorithm for block cipher algorithms. Arab J Sci Eng. 2024. doi: 10.1007/s13369-024-09423-6

[64] Razaq A, Maghrabi LA, Ahmad M, Aslam F, Feng W. Fuzzy logic-based substitution-box for robust medical image encryption in telemedicine. IEEE Access. 2024;12:7584–608. doi: 10.1109/access.2024.3351794

[65] Verma V, Kumar S. Quantum image encryption algorithm based on 3D-BNM chaotic map. Nonlinear Dyn. 2024;113(4):3829–55. doi: 10.1007/s11071-024-10403-6

[66] Olvera-Martinez L, Cedillo-Hernandez M, Diaz-Rodriguez CA, Faustinos-Morales L, Cedillo-Hernandez A, Garcia-Ugalde FJ. Symmetric grayscale image encryption based on quantum operators with dynamic matrices. Mathematics. 2025;13(6):982. doi: 10.3390/math13060982

[67] Zhu X-Y, Hu L-L, Gong L-H. Visually secure medical image encryption algorithm based on improved YOLOv11 and replacement strategy for region of interest. Nonlinear Dyn. 2026;114(2). doi: 10.1007/s11071-025-11972-w

[68] Duan Y, Xu X-Y, Banerjee S, Cao Y, Mou J. A multi-image encryption scheme for gray-color images based on 2D chaotic map and dual S-boxes. Nonlinear Dyn. 2026;114(4). doi: 10.1007/s11071-025-12116-w

