# Supplementary material for: A two-stage algebraic framework for designing cryptographically strong S-boxes for secure image encryption
Source: PLoS One. 2026 Sep 15;21(9):e0357107. doi: 10.1371/journal.pone.0357107 (PMC13577422; doi:10.1371/journal.pone.0357107)
Supplement: Supplementary file 1 [file pone.0357107.s001.docx]

**S1 Appendix. Positional action of the Stage 2 refinement permutation on the initial S-box.**

In Step 2 of the proposed S-box construction, we identified that applying the permutation $g=a^{75}b^{95}c^{11}d^{23}e^{2}$ to the initial S-box produces the final S-box with the highest nonlinearity score of $112$. This appendix illustrates how this transformation is carried out in terms of element positions.

For this positional illustration, the 256 entries of the S-box are numbered from 1 to 256 in row-major order, while the S-box values themselves remain in the range 0 to 255.

The generating permutations are:

*a=(1,96,103,24,221,133,136,42,25,113,12,40,248,119,26,112,159,18,222,5,255,124,90,150,154,91,126,157,80,237,243,176,98,215,97,54,192,58,106,63,183,137,204,228,45,48,99,60,234,166,95,217,15,72,230,211,110,20,9,172,238,2,4,85,162,53,43,220,65,116,8,208,73,51,57,165,214,66,37,94,49,77,171,190,109,21,107,125,245,209,152,242,142,121,182,75,201,38,132,29,224)*

*b=(3,86,218,114,88,105,170,62,120,19,184,253,129,179,194,44,198,27,181,193,177,35,87,70,205,186,226,30,46,128,145,207,191,23,175,111,144,64,140,247,196,134,102,76,229,71,22,47,41,212,50,163,69,180,189,52,36,199,115,200,11,6,246,195,135,92,131,93,158,161,155,241,7,169,68,151,256,188,28,32,55,149,82,185,164,225,227,108,138,236,118,216,139,14,160,100,146)*

*c=(10,206,33,219,187,240,84,231,178,143,197,17,39,250,213,233,249,203,254,61,202,101,13,79,16,31,74)*

*d=(34,81,89,59,122,117,244,252,123,147,83,174,130,173,210,167,168,239,104,232,156,251,148,141,223,67,56,153)*

and

*e = (78,235,127).*

For the element $g=a^{75}b^{95}c^{11}d^{23}e^{2}$, these powers yield the following combined permutation:

$a^{75}b^{95}c^{11}d^{23}e^{2}$*=(1,165,166,150,132,73,99,255,75,116,228,18,142,43,183,26,209,85,58,40,107,238,97,25,190,20,176,133,49,230,80,103,66,217,91,224,57,234,90,38,208,48,5,182,65,204,159,242,53,63,119,245,4,192,12,21,172,215,42,171,110,243,221,94,72,157,96,214,95,154,29,51,60,124,201,8,45,222,121,220,137,112,152,162,106,248,125,2,54,113,109,9,98,136,77,211,237,24,37,15,126)(3,100,14,216,236,108,225,185,149,32,188,151,169,241,161,93,92,195,6,200,199,52,180,163,212,47,71,76,134,247,64,111,23,207,128,30,186,70,35,193,27,44,179,253,19,62,105,114,86,146,160,139,118,138,227,164,82,55,28,256,68,7,155,158,131,135,246,11,115,36,189,69,50,41,22,229,102,196,140,144,175,191,145,46,226,205,87,177,181,198,194,129,184,120,170,88,218)(10,17,13,84,203,206,39,79,231,254,33,250,16,178,61,219,213,31,143,202,187,233,74,197,101,240,249)(34,141,104,173,123,59,56,251,168,174,244,81,223,232,210,147,122,153,148,239,130,252,89,67,156,167,83,117)(78,127,235).*

Here, each cycle specifies how positions of the S-box entries are shifted:

- In the cycle $(1,165,166,150,132,\ldots,37,15,126),$ the element at position 1 in the initial S-box moves to position 165, the element at position 165 moves to position 166, the element at position 166 moves to position 150, and this process continues until the element at position 126 moves back to position 1.
- In the cycle $(3,100,14,216,236,\ldots,170,88,218),$the element at position 3 moves to position 100, the element at position 100 moves to position 14, the element at position 14 moves to position 216, and the same procedure continues until the element at position 218 moves back to position 3.

All other cycles are applied in the same manner, moving elements according to the positions specified in each cycle. This iterative process continues for all mappings, thereby constructing the proposed S-box.
